# Systematic comparison and prediction of the effects of missense mutations on protein-DNA and protein-RNA interactions

**DOI:** 10.1371/journal.pcbi.1008951

**Published:** 2021-04-19

**Authors:** Yao Jiang, Hui-Fang Liu, Rong Liu

**Affiliations:** Hubei Key Laboratory of Agricultural Bioinformatics, College of Informatics, Huazhong Agricultural University, Wuhan, P. R. China; Queen’s University, CANADA

## Abstract

The binding affinities of protein-nucleic acid interactions could be altered due to missense mutations occurring in DNA- or RNA-binding proteins, therefore resulting in various diseases. Unfortunately, a systematic comparison and prediction of the effects of mutations on protein-DNA and protein-RNA interactions (these two mutation classes are termed MPDs and MPRs, respectively) is still lacking. Here, we demonstrated that these two classes of mutations could generate similar or different tendencies for binding free energy changes in terms of the properties of mutated residues. We then developed regression algorithms separately for MPDs and MPRs by introducing novel geometric partition-based energy features and interface-based structural features. Through feature selection and ensemble learning, similar computational frameworks that integrated energy- and nonenergy-based models were established to estimate the binding affinity changes resulting from MPDs and MPRs, but the selected features for the final models were different and therefore reflected the specificity of these two mutation classes. Furthermore, the proposed methodology was extended to the identification of mutations that significantly decreased the binding affinities. Extensive validations indicated that our algorithm generally performed better than the state-of-the-art methods on both the regression and classification tasks. The webserver and software are freely available at http://liulab.hzau.edu.cn/PEMPNI and https://github.com/hzau-liulab/PEMPNI.

## Author summary

Protein-nucleic acid interactions play important roles in various cellular processes. Missense mutations occurring in DNA- or RNA-binding proteins (termed MPDs and MPRs, respectively) could change the binding affinities of these interactions. Previous studies have compared protein-DNA and protein-RNA interactions from multifaceted viewpoints, but less attention has been given to the similarities and specific differences between the effects of MPDs and MPRs and between the methodologies for predicting the affinity changes induced by the two mutation classes. Therefore, we systematically compared their impacts and demonstrated that MPDs and MPRs could have specific preferences for binding affinity changes. These observations motivated us to construct regression models separately for MPDs and MPRs by introducing novel energy and nonenergy descriptors. Although similar frameworks were developed to estimate these two categories of mutation effects, different descriptors were selected in the regression models and further revealed the specificity of mutation classes. The interplay between the energy and nonenergy modules effectively improved prediction performance. Our algorithm can also be adopted to disentangle mutations significantly decreasing binding affinities from other mutations.

## Introduction

Protein-nucleic acid interactions (PNIs) play highly important roles in a variety of biological processes. For instance, protein-DNA interactions (PDIs) are central to DNA replication, repair, and recombination, whereas protein-RNA interactions (PRIs) are indispensable for post-transcriptional regulation and protein synthesis [1–4]. The appropriate binding affinities for these two types of interactions, which are largely determined by diverse intermolecular forces and structural properties, are crucial for the fulfillment of biological processes. Missense mutations occurring in DNA- or RNA-binding proteins could alter the above determinants, therefore leading to changes in binding free energies [5,6]. Furthermore, these alterations are closely associated with various diseases, such as cancer [7–10]. Quantifying the impacts of mutations on PNIs is useful in uncovering the pathogenesis of diseases and providing rational therapeutic strategies. To date, different experimental techniques, including isothermal titration calorimetry, surface plasmon resonance, and fluorescence resonance energy transfer, have been utilized to measure the binding free energies between proteins and nucleic acids [11–13], but these experimental measurements are time-consuming and labor-intensive and cannot satisfy the explosive growth of genomic data. Accordingly, it is highly desirable to establish complementary computational approaches for predicting binding affinity changes.

Over the past several years, a series of computational studies have been devoted to the effects of mutations on PDIs and PRIs (these two mutation classes are termed MPDs and MPRs, respectively). From the viewpoint of data curation, Prabakaran et al. first constructed a database named ProNIT that included experimental thermodynamic data regarding protein-nucleic acid binding [14]. Recently, Liu et al. established the dbAMEPNI database through manually collecting alanine mutagenesis data for PNIs [15]. Ramos and Moreira performed computational alanine scanning mutagenesis on protein-DNA complexes through the molecular mechanics/Poisson–Boltzmann surface area (MM/PBSA) and molecular mechanics/generalized Born surface area (MM/GBSA) methods [16]. Based on the MPD and MPR data extracted from ProNIT, Pires et al. developed the mCSM-NA algorithm that predicted the binding affinity changes using graph-based signatures in conjunction with pharmacophore modeling [17]. Peng et al. introduced a linear regression-based method called SAMPDI to estimate the energy changes of PDIs based on the modified MM/PBSA approach and several knowledge-based descriptors [18]. Zhang et al. proposed the PremPDI algorithm, which adopted molecular mechanics force fields and fast sidechain optimization methods to yield the inputs of multiple linear regression models for MPDs [19]. Additionally, several studies have combined machine learning methods with structural and sequence properties to identify hotspots in PDIs and/or PRIs from an energy perspective [20–22]. Collectively, these works provided novel insights into the understanding and prediction of protein-nucleic acid binding affinity changes.

Despite the significant progress mentioned above, a number of issues in this field have yet to be investigated. First, the effects of MPDs and those of MPRs remain to be systematically compared. It is still unclear whether there are differences in the trends of the energy changes upon mutations between these two types of interactions. Second, most existing algorithms were established based on MPD data, probably due to the scarcity of experimental data for MPRs [16,18,19]. Few works have assessed whether a unified computational framework can be used effectively for both MPDs and MPRs. In other words, can the existing methodology for MPDs be directly extended to MPRs? Third, the current energy-based prediction methods mainly used the energies of whole complexes as descriptors [18]. Intuitively, the energies of different geometric partitions in the complexes would also be associated with the binding affinity changes, which might prompt us to design novel energy descriptors, but less attention has been given to this point. Fourth, knowledge-based features were also adopted in previous works and integrated with energy features by linear combination models [18,19]. However, a comprehensive comparison of the utilities of energy and nonenergy features is lacking. Furthermore, the best way to combine them to improve prediction performance has yet to be explored.

Inspired by these problems, we attempted to perform a systematic comparison and prediction of the effects of MPDs and MPRs in this study. To this end, we first collected high-quality data for both mutation classes from existing resources and compared their impacts from different perspectives. Then, we designed novel energy- and nonenergy-based descriptors and evaluated their performance on MPDs and MPRs, respectively. Furthermore, we applied feature selection techniques to these two groups of features, thereby yielding the optimal energy- and nonenergy-based regression models for each type of mutation. Considering the interplay between the individual models, we built an integrative algorithm called PEMPNI (Predictors for Effects of Mutations on PNIs) for improving prediction performance. Further, this strategy was extended to the prediction of mutations significantly decreasing binding affinities. Collectively, this work provides an effective computational framework to analyze and predict the impacts of MPDs and MPRs, which may advance our understanding of the mechanism of PNIs.

## Materials and methods

### Dataset collection

**Fig 1:**
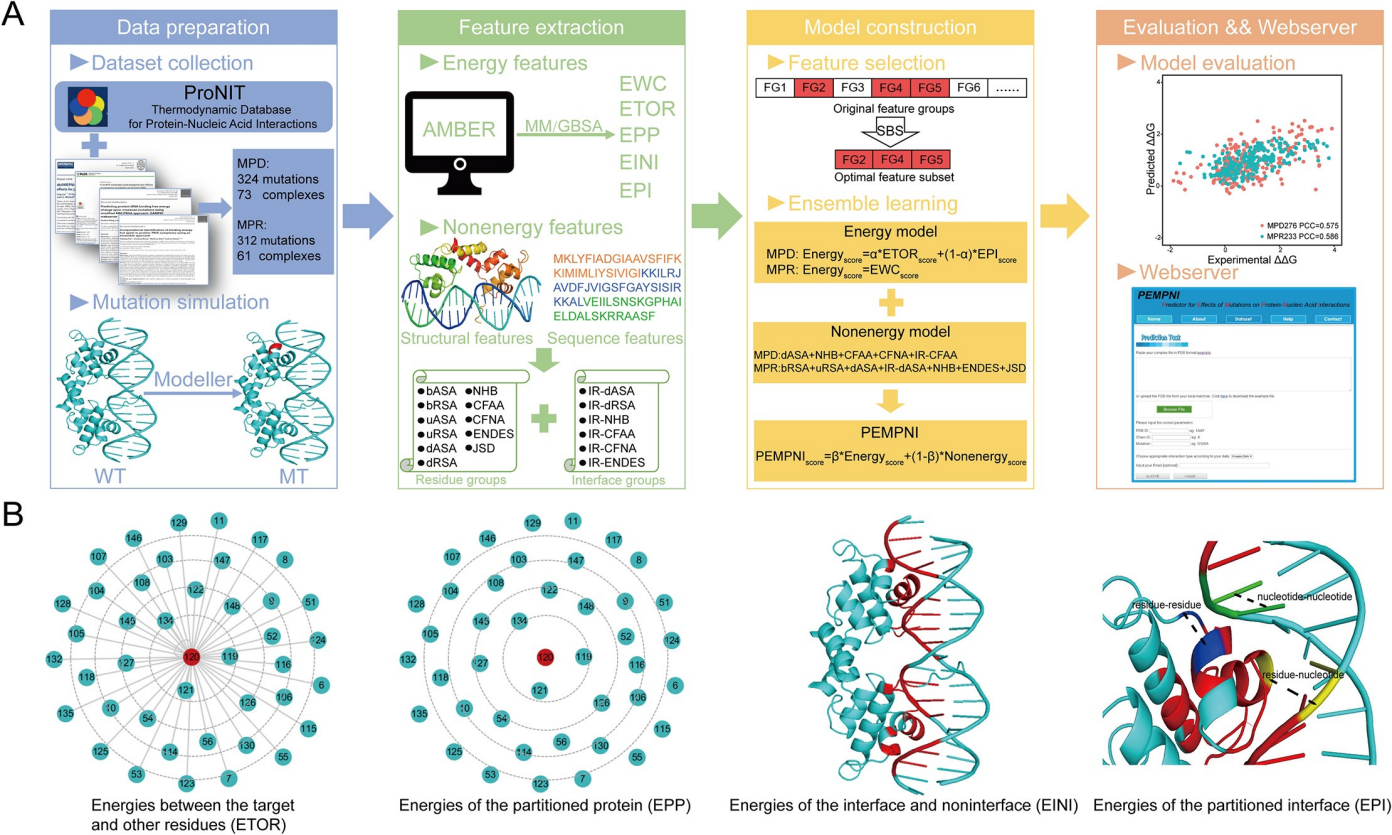
Schematic illustration of PEMPNI and novel energy features. (A) Flowchart of our computational framework. (B) Geometric partition-based energy features.

As shown in Fig 1A, the experimentally measured binding free energy changes upon mutations in protein-nucleic acid complexes were collected from several well-established databases (or datasets) and associated references. We downloaded a total of 11608 mutations from ProNIT and filtered out the records using the following criteria [14]. First, we removed the entries without structural information, the entries missing energy measures for wild-type or mutant sites, and the entries including nucleotide mutations or multiple residue mutations. Second, we compared the protein and nucleic acid sequences in ProNIT with the corresponding chains extracted from the complex structures using an in-house program. Mutations that could not be matched to residues in the PDB file were eliminated. We only reserved the entries possessing greater than 80% sequence identity for both the whole sequences and the binding sites in nucleic acids (i.e., the sequence fragment involving all interface nucleotides). Third, when a mutation corresponded to multiple energy measures in terms of different experimental conditions, we preferentially chose the measure generated from the isothermal titration calorimetry method along with the temperature and ion concentration of approximately 25°C and 155 mM. When all the multiple measures for a mutation were provided by other experimental methods or conditions, we might refer to additional data resources, including the dbAMEPNI database and the datasets of three existing methods (i.e., SAMPDI, PremPDI, and PrabHot) [15,18,19,21]. If the identical record was found, the corresponding measure was selected. Using the above procedures, we retained 154 mutations from 21 complexes. Furthermore, we gathered the mutations collected by dbAMEPNI, SAMPDI, PremPDI, and PrabHot. By manually checking the associated references, we compared the reported data with the PDB records according to the above criteria and collected the samples that were not included in the above four resources, which yielded 482 additional mutations. Finally, we obtained 324 mutations from 73 protein-DNA complexes and 312 mutations from 61 protein-RNA complexes. For MPDs, because our method was to be compared with other approaches (i.e., SAMPDI, PremPDI, and mCSM-NA), we selected 48 mutations from 20 complexes (MPD48) that were not used by the three competing algorithms for independent testing and the remaining 276 mutations from 53 complexes (MPD276) for training our models. For MPRs, we chose 79 mutations from 14 complexes (MPR79) prepared by mCSM-NA as the test set and the other 233 mutations from 47 complexes (MPR233) as the training set. A summary of our datasets is provided in S1 Table. All datasets used in this work could be downloaded from the PEMPNI website.

### Structure optimization and energy calculation

Based on the wild-type complex structures, we generated corresponding mutant complex structures by using the Modeller program [23]. Each complex was processed using AMBER16 combined with the ff14SB and parmbsc1 force fields [24,25]. The *tleap* module was used to embed each complex into a TIP3P water box that extended 10 Å from any solute atom. This system was neutralized by adding counter ions (i.e., Na+ and Cl-). As suggested by Chen et al. [26], the preprocessed complex structure was optimized in three steps. First, we performed 5000 iterations of energy minimization by restraining protein backbones with an elastic constant of 50 kcal·mol^−1^·Å^−2^. Second, we continued to perform 5000 iterations through reducing the elastic constant to 10 kcal·mol^−1^·Å^−2^. Third, the structure was minimized for 5000 iterations without any constraint. In each step, the 5000 iterations included 2000 iterations of steepest descent minimization along with 3000 iterations of conjugate gradient minimization. The cutoff for generating the short-range interactions was 10 Å, while the long-range electrostatic interactions were processed using the particle mesh Ewald algorithm [27]. Based on the minimized structure in the last iteration, we used the MM/GBSA approach to calculate the binding free energy of wild-type and mutant complexes, which could be roughly divided into four energy terms as follows:

$$\Delta G\approx \Delta E_{ele}+\Delta E_{vdw}+\Delta G_{GB}+\Delta G_{SA}$$ (1)

where ΔE_ele_ denotes the electrostatic energy, ΔE_vdw_ denotes the van der Waals energy, and ΔG_GB_ and ΔG_SA_ are the polar and nonpolar components of the solvation energy, respectively. ΔE_ele_ and ΔE_vdw_ were calculated using the *sander* program. ΔG_GB_ was estimated based on five different GB models, including GB^HCT^, GB^OBC1^, GB^OBC2^, GB^GBn1^, and GB^GBn2^ [28–31]. ΔG_SA_ was calculated using the LCPO algorithm, in which the solvent accessible surface area was the determinant [32]. For the binding free energy and each energy term, the total value could be decomposed at the residue pair level by running the *MMPBSA* program. We also tested the MM/PBSA approach in the initial stage but abandoned this strategy due to its higher computational costs and relatively lower performance.

### Energy-based features

Based on the above procedure, we obtained five energy descriptors (i.e., ΔG, ΔE_ele_, ΔE_vdw_, ΔG_GB_, and ΔG_SA_) for each complex (termed the energies of the whole complex, EWC) and residue pairs in this complex. Previous studies mainly adopted the energy terms of complexes as descriptors to predict the binding affinity changes. As illustrated in Fig 1B, herein, we attempted to design novel characteristics to measure the energy contributions of different partitions of each complex based on the decomposed energies for residue pairs. First, we computed the energies between the mutated residue and other residues in a specific partition of the protein according to the distances between residues, such as 0 Å (i.e., the target residue itself), 0~3 Å, 3~4 Å, 4~5 Å, 5~6 Å, and >6 Å (termed the energies between the target and other residues, ETOR). Second, based on the aforementioned geometric partitions, we calculated the sum of residue energies in each partition (termed the energies of the partitioned protein, EPP). The energy of each residue was the total value of interaction energies between this residue and all residues and nucleotides in the complex. Third, each complex was separated into two partitions, namely, the interface and noninterface regions. A residue-nucleotide physical contact was formed if the distance between one heavy atom of a residue and that of a nucleotide was less than 5 Å. We calculated the sum of pairwise energies in each partition (termed the energies of the interface and noninterface, EINI). Regarding the former, all pairwise combinations among the residues and nucleotides in a given interface (see below) were considered, and the remaining pairs were categorized into the latter. Fourth, all the pairs involved in an interface were further divided into three types, including residue-residue pairs, residue-nucleotide pairs, and nucleotide-nucleotide pairs. The total energies were computed for these three sections (termed the energies of the partitioned interface, EPI). The novel feature groups could be presented as follows:

$${ETOR}_{i}=\sum_{{o\in PT}_{i}}{pairwise\_energy}_{to}$$ (2)

$${EPP}_{i}=\sum_{{o\in PT}_{i}}{residue\_energy}_{o}$$ (3)

$${EINI}_{i}\;or\;{EPI}_{i}=\sum_{{m,n\in PT}_{i}}{pairwise\_energy}_{mn}$$ (4)

where *t* and *o* denote the target and other residues, respectively, and *m* or *n* denotes one residue or nucleotide in a given partition. *i* denotes the index of a partition (*PT*), and the partitions of each group have been mentioned above (S2 Table).

### Nonenergy-based features

In addition, we extracted other structural and sequence features, including solvent accessibilities, hydrogen bonds, contact features, ENDES features, and evolutionary conservation. Unlike previous studies that mainly focused on the structural attributes of mutated residues, we hypothesized that changes in the binding free energy of complexes could be associated with alterations in the structural properties of interfaces, therefore extracting the corresponding features for interface residues.

#### Solvent accessibilities

We used the NACCESS program to compute the absolute and relative accessible surface area (ASA and RSA) of each residue according to total atoms, mainchain atoms, sidechain atoms, polar sidechain atoms, and nonpolar sidechain atoms [33]. For a target residue, we calculated its ASA and RSA in the bound and unbound states (bASA, bRSA, uASA, and uRSA), respectively. The differences in solvent accessibilities between the two states (dASA and dRSA) were also extracted. Further, we computed the cumulative dASA and dRSA values for interface residues, therefore yielding the IR-dASA and IR-dRSA features.

#### Hydrogen bonds

The number of hydrogen bonds between the target residue and the remainder of a complex (NHB) was computed using the HBPLUS program [34]. We also calculated the cumulative NHB of total residues in each interface (IR-NHB).

#### Contact features

The contact features included two descriptors, namely, the residue-residue contact strength and the average atomic contact strength. The first descriptor was the number of residues interacting with the target residue, and the second descriptor was the ratio of the number of all atomic contacts between the target and its interacting residues to the number of interacting residues. For each target residue, we computed these features in terms of the interactions involving amino acids and nucleic acids (CFAA and CFNA), respectively; the corresponding features for an interface were named IR-CFAA and IR-CFNA.

#### ENDES features

The ENDES descriptors that were originally designed for protein-protein docking included seven knowledge-based scores, such as the sidechain score, propensity score, and conservation score [35]. We calculated these measures for each target residue and all interface residues (ENDES and IR-ENDES), respectively.

#### Evolutionary conservation

The evolutionary conservation of each residue was denoted by the Jensen-Shannon divergence (JSD), which was calculated based on the weighted observed percentages generated by running three iterations of PSI-BLAST with an *e*-value cutoff of 0.001 [36,37].

### Feature selection, model construction and performance evaluation

Regarding each mutation, we can generate five energy feature groups using a given GB model (S2 Table) and 17 nonenergy feature groups at the structural and sequence levels (S3 Table). Aside from the features extracted from the wild-type complex, the differences in measures of the native and mutant complexes were also used in this work. As shown in Fig 1A, we considered each feature group as the basic unit and utilized the sequential backward selection (SBS) algorithm to generate the optimal combination of energy or nonenergy feature groups. Specifically, the SBS procedure started with all feature groups and assessed their performance by the Pearson correlation coefficient (PCC). We iteratively deleted a feature group so that the remaining feature groups could improve prediction performance to the greatest extent possible. This process was halted if the PCC value failed to increase. Feature selection was performed on the MPD276 and MPR233 datasets. From the viewpoint of energy, the ETOR and EPI groups were reserved for MPDs, whereas only the EWC group was selected for MPRs. For MPDs, moreover, we found that the weighted result of the outputs from the ETOR- and EPI-based models was superior to the result based on the direct integration of both feature groups. Thus, formula (5) was adopted for energy feature-based prediction. Regarding the nonenergy-based models, five feature groups (i.e., dASA, NHB, CFAA, CFNA, and IR-CFAA) were reserved for MPDs, and seven groups (i.e., bRSA, uRSA, dASA, IR-dASA, NHB, ENDES, and JSD) were selected for MPRs. As revealed in formula (6), we considered their integrative output as the final prediction value through the interplay between energy- and nonenergy-based models.

$$\left\{ \begin{array}{l} MPD:\;Energy_{score}=\alpha *{ETOR}_{score}+(1-\alpha )*{EPI}_{score}\\ MPR:\;Energy_{score}={EWC}_{score} \end{array}\right.$$ (5)

$${PEMPNI}_{score}=\beta *Energy_{score}+(1-\beta )*{Nonenergy}_{score}$$ (6)

where *α* was set to 0.4 when using GB^HCT^ and GB^GBn2^ and 0.5 when using the other three GB models. The GB^HCT^ and GB^OBC1^ models were finally selected for MPDs and MPRs, respectively. *β* was set to 0.6 and 0.5 for the two mutation classes, respectively. To obtain the optimal parameters, we examined different *α* and *β* values in the range of 0 to 1 with a step of 0.1 (S1 Fig). S4 Table shows the parameters used in our models.

We implemented the regression models using the random forest regressor provided by the scikit-learn package [38]. To assess prediction performance, we conducted leave-one-complex-out validation (LOCOV) on training sets. Specifically, in each iteration, the mutations from one complex were used for testing, while the remaining mutations were used for training. Independent testing was also used to examine the generalization ability of our method. In addition to the PCC value, the root-mean-square error (RMSE) was reported for each model.

### The PEMPNI webserver

To facilitate the use of our algorithm, we implemented PEMPNI as an easy-to-use webserver, which is freely available at http://liulab.hzau.edu.cn/PEMPNI. Users could submit the protein-nucleic acid complex structure in PDB format and designate the mutated residue in proteins and the appropriate prediction model. The execution time is approximately 2 to 3 hours for each submission because PEMPNI combines explicit solvent models and long-step minimizations to optimize complex structures. Once the calculation is finished, PEMPNI returns a result page that includes the predicted binding affinity change value and the predicted score of significantly decreasing binding affinity for the mutated residue. Furthermore, PEMPNI uses the JSmol software to display the wild-type and mutant complex structures, which could also be downloaded by users for follow-up analyses.

## Results

### Comparison of the effects of MPDs and MPRs from different viewpoints

**Fig 2:**
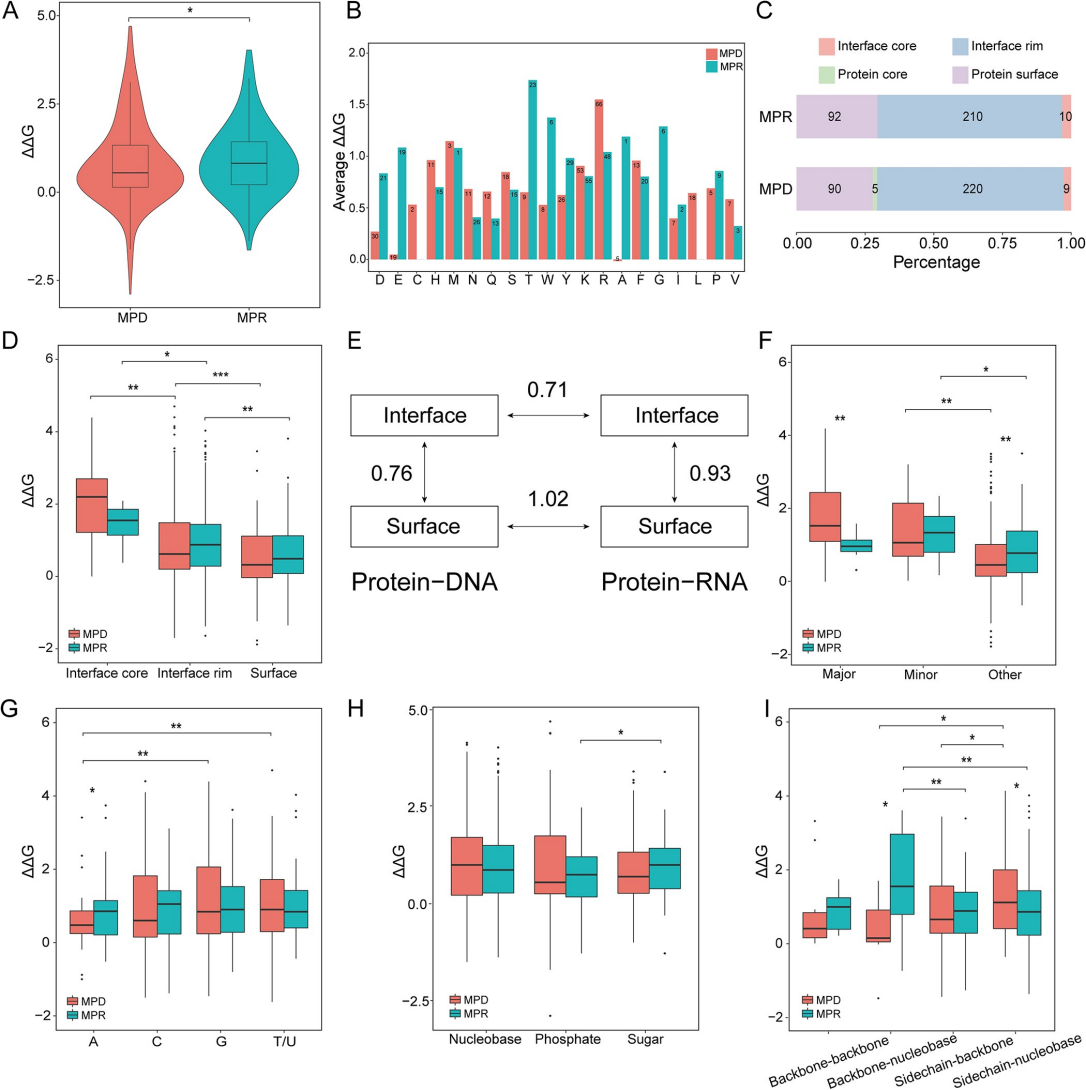
Comparison of binding affinity changes induced by MPDs and MPRs. (A) Overall comparison of MPDs and MPRs. (B) Comparison of MPDs and MPRs based on residue types. The numbers of mutations are shown in the columns. (C) Distribution of MPDs and MPRs in different geometric locations. (D) Comparison of MPDs and MPRs based on geometric locations. (E) Distances between different mutation groups. (F) Comparison of MPDs and MPRs interacting with major or minor grooves. (G-I) Comparison of MPDs and MPRs based on their major binding modes (i.e., the major interacting nucleotides (G), the major interacting subunits of nucleotides (H), and the major interacting contacts between the target residue and nucleotides (I)). For example, the major interacting nucleotides represent that the atomic contacts between a specific type of nucleotides and the target residue account for the greatest proportion among all contacts involving this residue. ^***^ P<0.001, ^**^ 0.001≤ P<0.01, and ^*^ 0.01≤ P<0.05.

Before developing the prediction models, we systematically compared these two categories of mutations collected in this work. From an overall perspective, as shown in Fig 2A, the energy changes induced by MPRs were relatively greater than those triggered by MPDs. In fact, compared to the mutations in single-stranded DNA- and RNA-binding proteins, those in double-stranded DNA-binding proteins had smaller influences, suggesting that the corresponding complexes were more stable (S2A Fig). In the wild-type structures, the positively charged residues (e.g., R, K, and H) that could interact with the negatively charged backbones of DNA contributed to more significant impacts on PDIs, while the negatively charged residues (e.g., D and E) yielded smaller affinity changes (Fig 2B). In contrast, it seems that no obvious rule could be found for PRIs. Based on the geometric locations defined by Levy et al. [39], we found that most mutated residues appeared in protein surfaces and interface rims, but both MPDs and MPRs from interface cores resulted in the greatest affinity changes, followed by those from interface rims, indicating that residues in the center of interfaces played highly important roles in stabilizing PNIs (Fig 2C and 2D). According to the average energy change of each residue type, we calculated the Euclidian distances between different groups using the formula: $\Delta d^{2}=\frac{1}{20}\sum_{i=1}^{20}{\left({\Delta \Delta G}_{i}-{\Delta \Delta G}_{i}^{\prime }\right)}^{2}$. As shown in Fig 2E, MPDs and MPRs from interfaces shared more similarities than these two classes from protein surfaces, and the differences between interfaces and surfaces were more remarkable for PRIs than for PDIs.

According to Barik et al.’s definition [40], we obtained 32 and 15 MPDs (9 and 14 MPRs) that physically interacted with the major and minor grooves of double-stranded DNA (RNA), respectively. In Fig 2F, MPDs contacting major grooves triggered greater changes, whereas MPRs associated with minor grooves generated relatively intensive impacts. Compared with the minor groove, the major groove of DNA is wide and more accessible, while the major groove of RNA is narrow and less accessible, so that the binding affinity of PDIs (PRIs) could be generally determined by major (minor) groove interactions [41]. For each mutation, moreover, we dissected its major binding mode based on the interacting nucleotides, the interacting subunits of nucleotides, and the interacting contacts between the target residue and nucleotides. As revealed in Figs 2G–2I and S2B-S2D, MPDs mainly interacting with T or G were able to exert stronger influences, while MPRs mainly forming contacts with phosphates could induce smaller impacts. Furthermore, MPDs mainly involved in sidechain-nucleobase contacts more easily destabilized PDIs, and MPRs mainly involved in backbone-nucleobase contacts affected PRIs more significantly. We thus proposed that the residues that were spatially closer to nucleic acids (e.g., residues interacting with nucleobases) were more important for the affinity of PNIs. For instance, the stability of PDIs is dependent on the formation of hydrogen bonds between protein sidechains and DNA bases [42]. Collectively, there were remarkable differences between the impacts caused by MPDs and MPRs, and the prediction models should thus be separately developed for these two mutation classes.

### Model construction and performance assessment based on LOCOV

**Fig 3:**
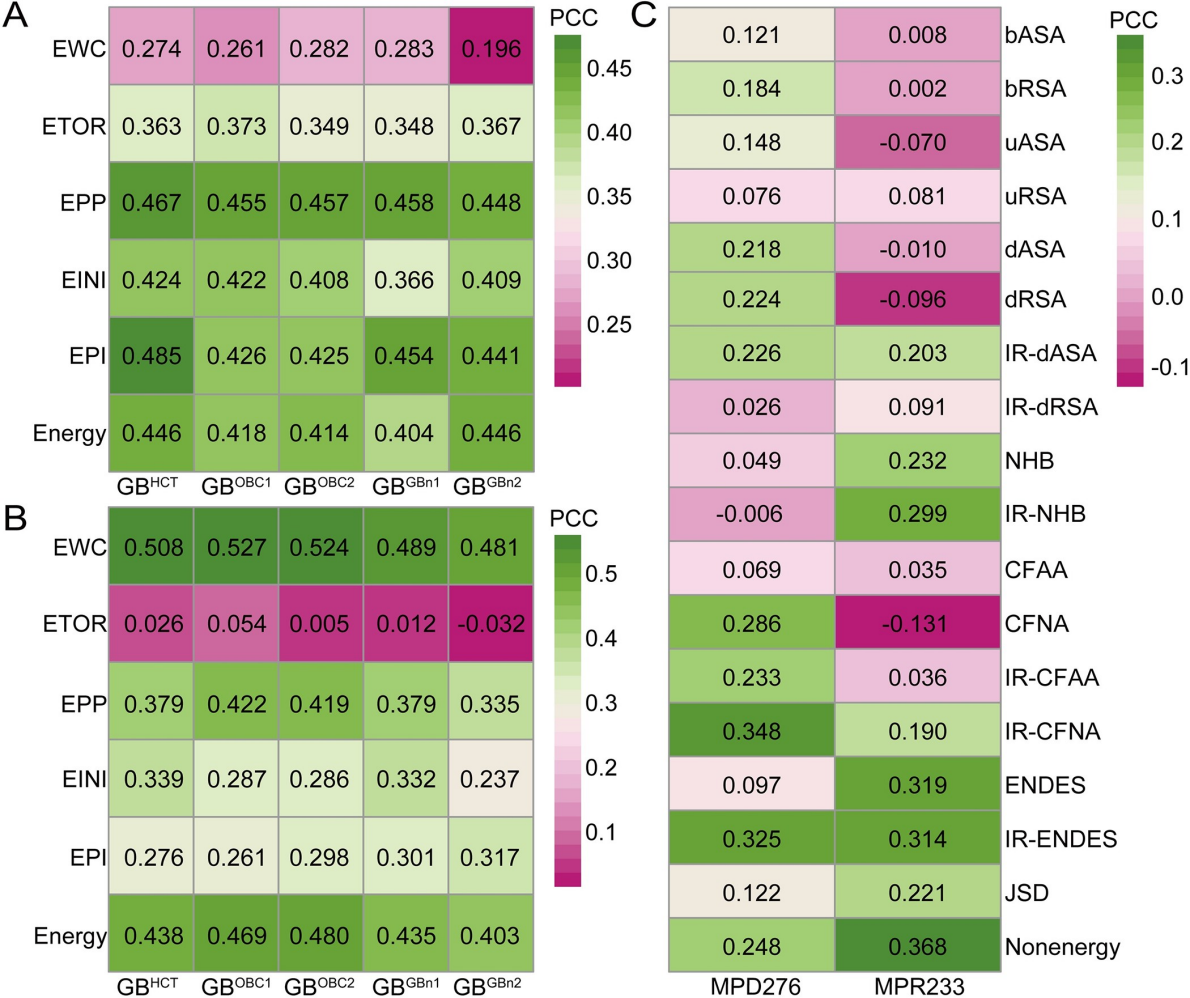
PCC values of individual and integrative feature groups for LOCOV. (A) PCC values of energy feature groups for MPD276. (B) PCC values of energy feature groups for MPR233. (C) PCC values of nonenergy feature groups for MPD276 and MPR233. The last row in each figure shows the performance of integrative feature groups.

We developed a group of regression models based on the individual feature groups and evaluated their performance on the MPD276 and MPR233 datasets by conducting the LOCOV procedure. As shown in Figs 3A and 3B and S3A and S3B, each energy feature group demonstrated a similar trend when using different GB models. From an overall perspective, the GB^HCT^- and GB^OBC1^-based energy groups exhibited the best results for MPD276 and MPR233, respectively. Regarding PDIs, the energy features derived from whole complexes (EWC) obtained the worst performance among the five feature groups. The EPI group performed most favorably, with a PCC and RMSE of 0.485 and 1.083, suggesting that the energies derived from the three separated partitions in protein-DNA interfaces could be closely associated with the binding affinity changes of PDIs. Regarding PRIs, in contrast, the EWC group remarkably outperformed the remaining four groups, with a PCC and RMSE of 0.527 and 0.855. This result suggested that the entire energies of complexes were extremely useful indicators to predict the effects induced by MPRs. Except for the ETOR group, every novel feature group revealed a certain degree of prediction ability for PRIs. Compared to the EWC group, moreover, the MM/GBSA total energy (i.e., *ΔG* in this group) achieved slightly better performance on MPD276 and remarkably worse performance on MPR233 (S5 Table). We also adopted different initial structures of mutant complexes generated from the Modeller program for energy minimizations and feature calculations. The results of three repetitions suggested that the selection of mutant structures had a trivial influence on prediction performance (S6 Table). Collectively, the energy features from different geometric partitions can be used to estimate the binding free energy changes and exhibit different tendencies for MPDs and MPRs.

As shown in Fig 3C, the performance of nonenergy feature groups was generally worse than that of energy feature groups. The IR-CFNA and ENDES groups achieved the highest PCC values of 0.348 and 0.319 for MPDs and MPRs, respectively. We also found that most feature groups could be used effectively for only one type of mutation. For instance, the ASA- and contact-related groups only demonstrated effectiveness for MPD276, while the hydrogen bond-related groups displayed preferences for MPR233. In contrast, the ENDES- and JSD-related groups provided useful information for both datasets. Moreover, the interface-based groups generally achieved better or comparable results compared to the corresponding target residue-based groups. We further checked whether these two classes of feature groups could synergistically improve the prediction results. S4 Fig illustrates that the use of combined groups based on structural features generated largely better performance, suggesting that interface-based groups could be treated as an effective complement to residue-based groups.

**Fig 4:**
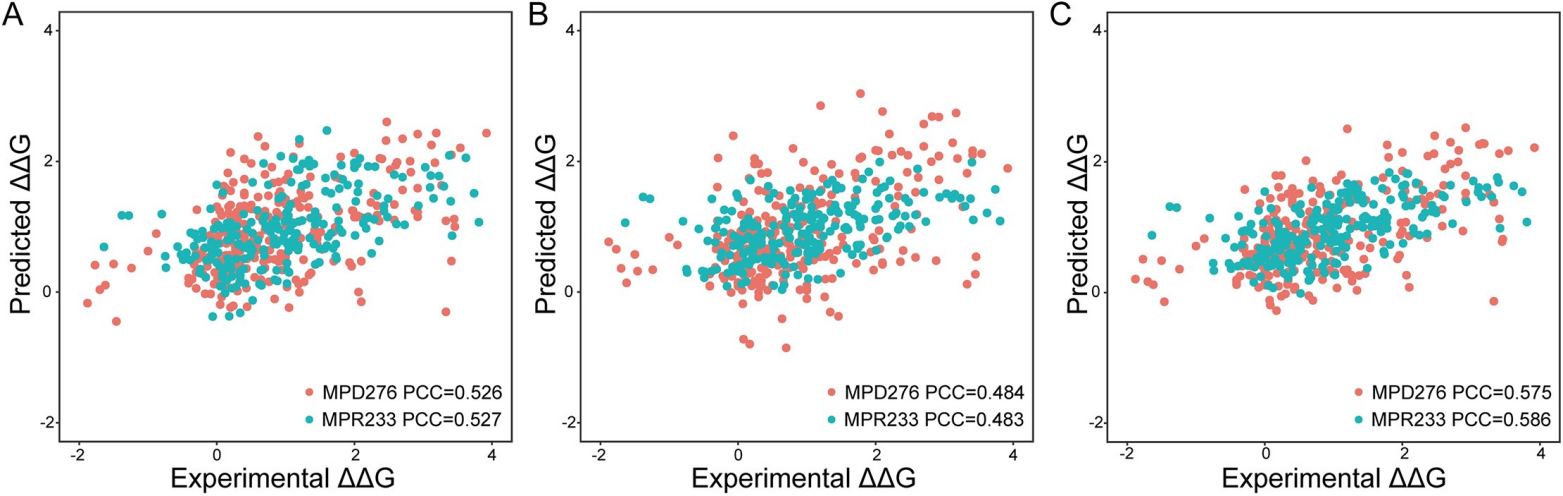
**PCC values of different models for LOCOV.** (A) Energy feature-based model. (B) Nonenergy feature-based model. (C) Integrative model (PEMPNI).

Afterwards, we combined all energy or nonenergy feature groups for constructing regression models. As shown in Fig 3, their results were even worse than the results of some individual groups, implying that redundant information affected the performance and should be eliminated. After performing the SBS procedure, the energy feature-based model obtained a PCC and RMSE of 0.480 and 1.068 for MPDs by reserving the EPI and ETOR groups. Furthermore, we examined the weighted sum of the predicted values from these two groups and obtained an improved PCC and RMSE of 0.526 and 1.014 (Figs 4A and S5). For MPRs, only the EWC group was reserved through feature selection. Regarding nonenergy-based models, we obtained a PCC and RMSE of 0.484 and 1.050 for MPDs through combining five feature groups, among which there were three contact-related groups. Four ASA-related groups, in contrast, together with the other three groups constituted the optimal feature subset for MPRs, which generated a PCC and RMSE of 0.483 and 0.882 (Fig 4B). The correlation of energy- and nonenergy-based predicted values implied that these two models may complement each other (S6 Fig). As shown in Fig 4C, the integrative algorithm yielded PCCs of 0.575 and 0.586 for MPDs and MPRs, along with RMSEs of 0.978 and 0.832. These results suggested that similar computational frameworks could be utilized to estimate the changes induced by both MPDs and MPRs, but the final reserved features reflected the specificity of these two types of mutations.

**Fig 5:**
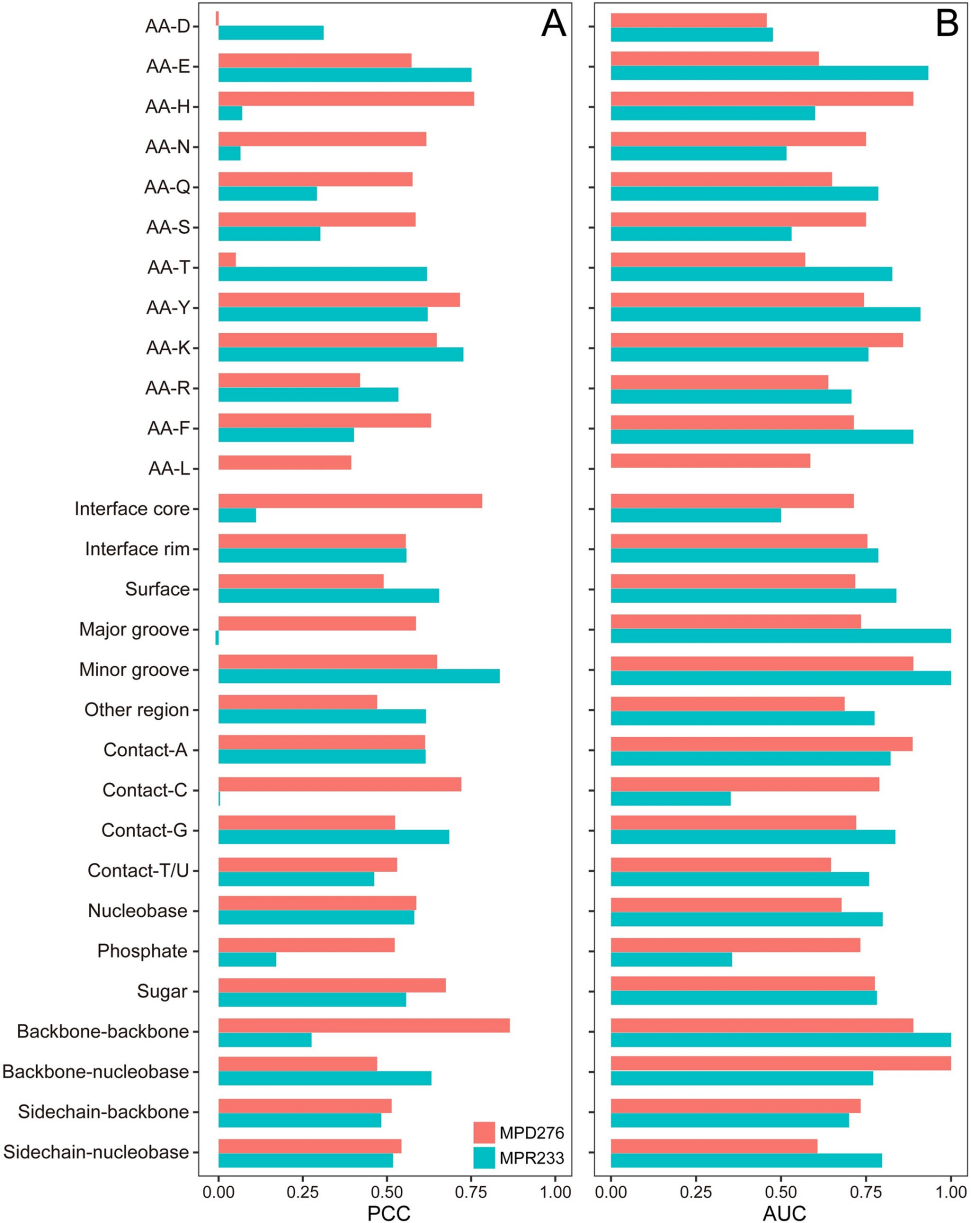
Performance of PEMPNI on different mutation subgroups. (A) PCC values for regression tasks. (B) AUC values for classification tasks. The subgroups that had a small number of samples were not included in this analysis.

After achieving optimal performance on MPD276 and MPR233, we divided each dataset into different subsets according to the first section of Results (Fig 5A). In terms of wild-type residues, PEMPNI showed the highest PCCs for H and Y from MPDs and for E and K from MPRs. In contrast, the PCC values for D and T from MPDs and those for H and N from MPRs were very poor. Regarding geometric locations, we obtained the highest correlation for MPDs from interface cores but the lowest measure for MPRs from the corresponding regions. Facing double-stranded binding partners, our method yielded a very low PCC for MPRs involved in major grooves but performed favorably on the remaining subsets. According to the major binding modes of mutated residues, only the MPR subset mainly interacting with cytosines or phosphates had a PCC value less than 0.2. Collectively, the performance of PEMPNI on various subsets was largely acceptable, but some MPR subsets were more challenging than their MPD counterparts. More importantly, these analyses indicated that the difficult samples should be given more concern in the future.

### Independent testing and comparison with existing methods

**Fig 6:**
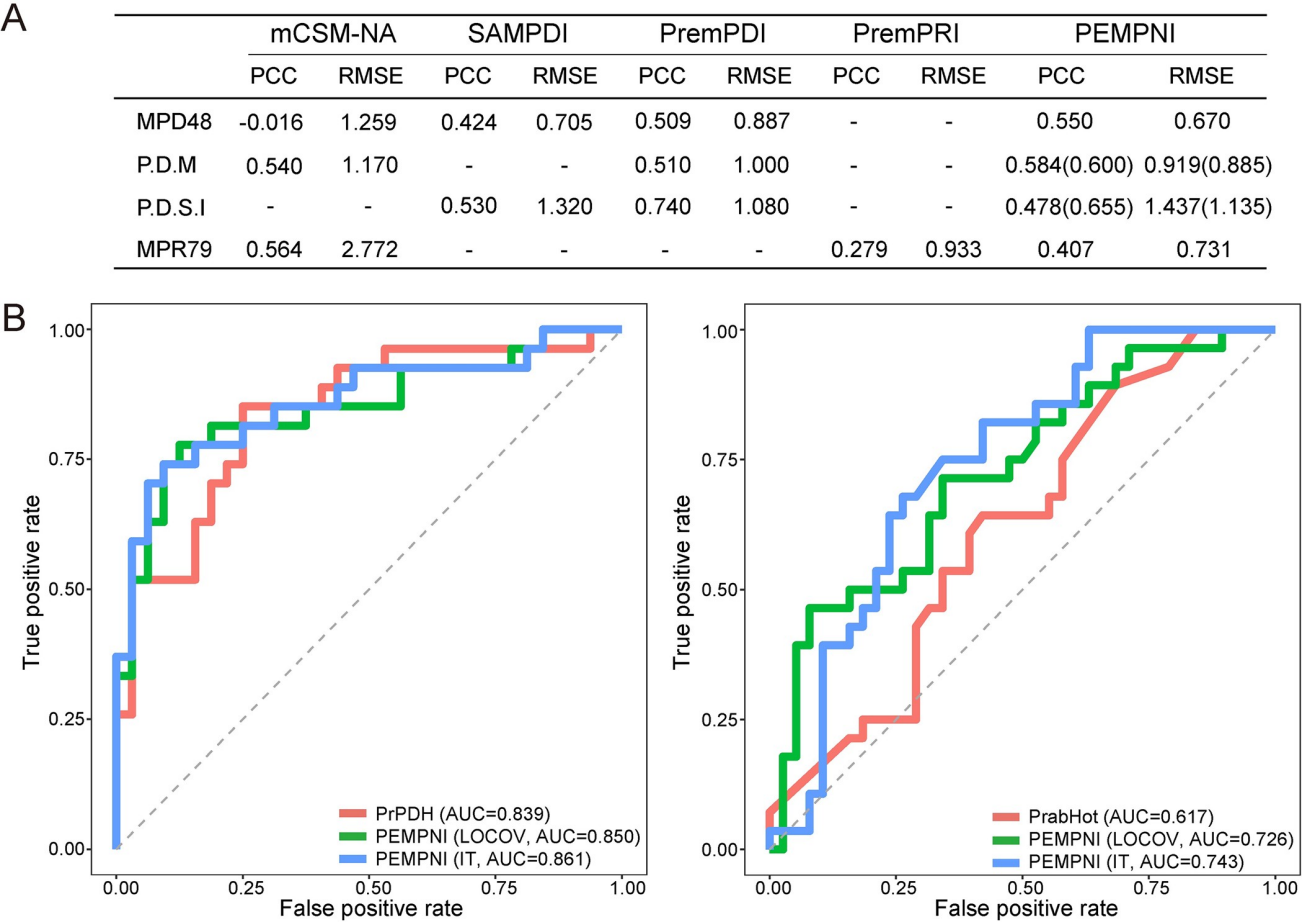
Comparison of PEMPNI and existing methods. (A) PEMPNI versus other methods for regression tasks. The performance in parentheses was generated from our algorithm trained by the datasets of PremPDI. (B) PEMPNI versus other methods for classification tasks.

To further examine the usefulness of our method, we evaluated the individual and integrative feature groups using the MPD48 and MPR79 datasets (S7 and S8 Figs). The P.D.M and P.D.S.I datasets prepared by Zhang et al. [19], which were originally adopted as test sets to compare PremPDI with mCSM-NA and SAMPDI, were also used to test the MPD-related predictors. P.D.M had 114 mutations from 33 complexes collected by PremPDI rather than mCSM-NA, and P.D.S.I had 77 interfacial mutations from 32 complexes collected by PremPDI rather than SAMPDI. PremPDI utilized the remaining 105 and 142 mutations as training sets, respectively. To evaluate PEMPNI, we excluded all overlapping complexes from our MPD datasets and retained 206 and 172 mutations for training, respectively (S9–S11 Figs). As shown in S12 Fig, our energy feature-based model obtained PCCs of 0.528, 0.606, 0.535, and 0.355 for the four test sets, while the nonenergy feature-based model performed relatively worse on these datasets except MPR79. Using the integrative predictions from these two models, we generated better or comparable measures, with PCCs of 0.550, 0.584, 0.478, and 0.407, respectively, indicating the interplay between two modules and the robustness of PEMPNI. Based on MPD48, we compared our method with existing algorithms, including mCSM-NA, SAMPDI, and PremPDI [17–19]. mCSM-NA used graph-based signatures to estimate the binding affinity changes, while SAMPDI and PremPDI were dependent on a linear combination of several energy- and structure-based features. As shown in Fig 6A, PEMPNI outperformed these three methods on MPD48 by both the PCC and RMSE measures. Compared with PremPDI, PEMPNI trained on our dataset could generate a higher PCC for P.D.M but a lower PCC for P.D.S.I. If we retrained our models using the same training data of PremPDI, these two methods possessed comparable PCCs and RMSEs. Additionally, we compared our method with mCSM-NA based on the MPR79 dataset. Although mCSM-NA generated a higher PCC value, its RMSE measure was approximately 3.5 times greater than our result. Moreover, we submitted MPR79 to the latest PremPRI webserver that was an extension of PremPDI for MPRs [43]. Although its training set included 31 overlapping mutations, PremPRI achieved worse PCC and RMSE metrics than our method. Overall, PEMPNI exhibited competitive performance compared to existing algorithms, which may be attributed to two reasons: (i) novel geometric partition-based energy features and interface-based structural features were included in this work; and (ii) energy- and nonenergy-based models were developed separately for each mutation class, and their interplay was fully used in the integrative framework.

### Prediction of mutations significantly decreasing binding affinities

It would be interesting to further examine whether our methodology could be used to discriminate mutations significantly reducing binding affinities (ΔΔG ≥ 1 kcal·mol^−1^) from other mutations. We thus replaced regression models with RF-based classification algorithms in our framework and evaluated their performance in terms of the Matthews correlation coefficient (MCC) and the area under the curve (AUC). As shown in S13 Fig, overall, the complementarity between novel energy feature groups and the synergy between energy- and nonenergy-based models were still effective for this classification problem. For instance, the combination of ETOR and EPI groups generated an AUC of 0.719 for MPD276, while the measures of the individual feature groups were 0.663 and 0.700, respectively. Further, by incorporating the nonenergy feature-based predictions, the AUC was elevated to 0.738. For MPR233, the integrative method performed more favorably than the EWC-based energy model and the nonenergy feature-based model, yielding an AUC of 0.793. As shown in Fig 5B, PEMPNI showed poor classification performance on the challenging subsets observed in the regression task. Regarding the four test sets, our method demonstrated better results for MPDs and moderately reduced measures for MPRs, indicating the generalization ability of different models (S13 Fig). We compared our algorithm with PrPDH and PrabHot, which were nonenergy feature-based approaches for predicting hotspots in protein-DNA and protein-RNA complexes, respectively [20,21]. Samples from MPD276 and MPR233 that were not in the training sets of PrPDH and PrabHot can be used as the benchmark. We obtained 59 mutations from 19 protein-DNA complexes and 66 mutations from 22 protein-RNA complexes. According to the LOCOV results, PEMPNI yielded AUCs of 0.850 and 0.726 for MPDs and MPRs, respectively (Fig 6B). If we adopted these samples for testing and used the remaining samples collected in this work for training, our algorithm obtained AUCs of 0.861 and 0.743. In contrast, PrPDH achieved an AUC of 0.839 for MPDs, and PrabHot generated an AUC of 0.617 for MPRs. These results illustrated the advantage of our integrative framework based on the energy and nonenergy modules.

### Case studies

**Fig 7:**
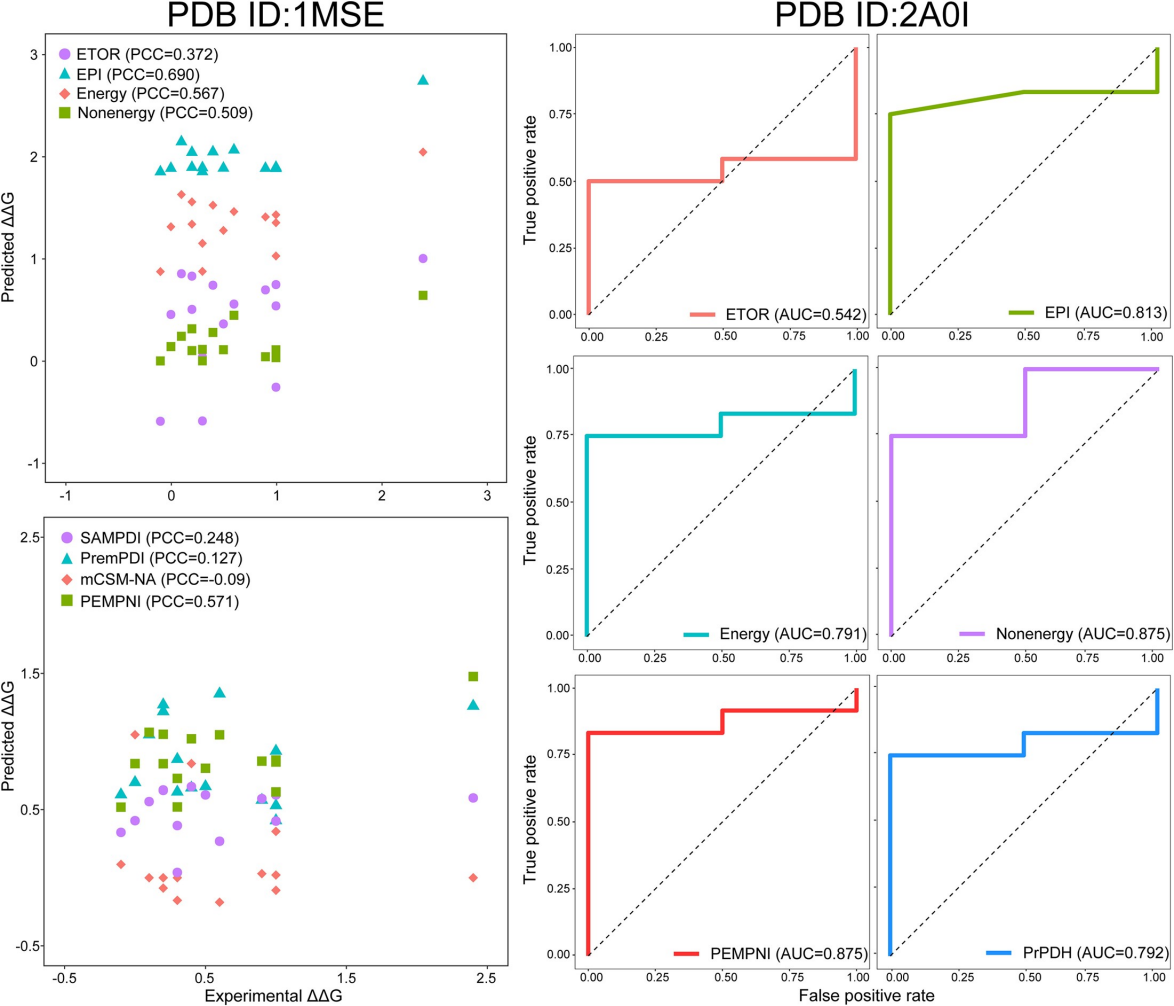
Performance of PEMPNI and other methods on representative protein-DNA complexes.

Finally, we selected several complexes as representatives to demonstrate the utility of the proposed framework. The MPD- and MPR-related examples were chosen from MPD276 and MPR233, respectively, and they generally contained a greater number of mutations that could be helpful in generating meaningful evaluation measures. These complexes were also found in the training sets of other methods. Notably, we showed the LOCOV results of PEMPNI for these samples but did not rigorously remove the possible bias of the competing algorithms (i.e., test samples were used for training). From the complex including the DNA-binding domain of Myb and its specific DNA sequence (PDB ID: 1MSE) [44], we obtained 15 MPDs to compare PEMPNI with three existing approaches. As revealed in Fig 7, the energies derived from the separated interface (EPI) made significant contributions to the energy-based prediction and even the final prediction results. The PCC measures of PEMPNI, PremPDI, SAMPDI, and mCSM-NA were 0.571, 0.127, 0.248, and -0.09, respectively. The second complex was the TraI protein of conjugative plasmid F factor binding to single-stranded DNA (PDB ID: 2A0I) [45], in which 14 mutations were available. The EPI group still played an important role in this example, but the nonenergy-based prediction model yielded more favorable measures. Our integrative algorithm outperformed PrPDH in terms of AUC (0.875 versus 0.792). The trp RNA-binding attenuation protein regulates the expression of the tryptophan biosynthetic genes of bacilli by interacting with single-stranded RNA (PDB ID: 1C9S) [46]. For the 15 MPRs in this complex, our energy- and nonenergy-based models achieved PCCs of 0.397 and 0.315, respectively (S14 Fig). Using their complementarity, PEMPNI obtained a higher PCC value than mCSM-NA (0.506 versus 0.250). The fourth example was the platform-PAZ-connector helix cassette of human Dicer in conjunction with small interfering RNAs (PDB ID: 4NGD) [47], which offered six MPRs for the comparison between PEMPNI and PrabHot. Although the energy-based model yielded very poor performance, the contribution of the nonenergy module prompted PEMPNI to achieve a greater AUC measure (0.625 versus 0.563). Based on these examples, we concluded that the integration of multifaceted information could generate more robust predictions and that PEMPNI could provide improved results in some cases compared to existing methods.

## Discussion

Both PDIs and PRIs are essential for biological processes. Previous computational studies have compared these two types of interactions and gained novel insights from multifaceted viewpoints, but less attention has been given to the similarities and specific differences between the effects of MPDs and MPRs and between the methodologies for predicting the energy changes induced by the two mutation classes. To fill this gap, we systematically investigated their impacts and demonstrated that MPDs and MPRs could induce similar tendencies for energy alterations according to their geometric locations but displayed specific preferences in terms of residue types and major binding modes. Afterwards, we designed novel geometric partition-based energy features and interface-based structural descriptors. By performing feature selection, we found that the energies between the mutated site and other residues together with the energies of separated interface regions were major determinants of the effects induced by MPDs, whereas the energies of whole complexes played a crucial role in predicting the impacts of MPRs. Regarding nonenergy feature-based models, the contact- and ASA-related features were important for PDIs and PRIs, respectively. The complementarity between energy- and nonenergy-based modules prompted us to construct an integrative algorithm that could improve prediction results. Furthermore, we demonstrated that our methodology was helpful in disentangling mutations significantly decreasing binding affinities from other mutations. Extensive evaluations indicated that PEMPNI largely performed better than other methods on both the regression and classification tasks. Finally, we implemented our algorithm as a user-friendly webserver (S15 Fig).

Despite the progress achieved here, several limitations in the present work should be noted for the future. First, although we adopted extensive validations to evaluate our method, the datasets of both mutation types were small. In addition to manual collection, advanced text mining techniques could be used to extract more data from the literature for the assessment and improvement of our method. Second, we used the feature groups as units to perform feature selection in this study because our initial experiments at the feature level showed that the resulting features were sensitive to different datasets. This problem would be solved when more abundant and diverse mutations become available. Third, we combined explicit solvent models and long-step minimizations to generate optimized structures, which required a relatively longer computation time. The energy minimization protocol could be optimized by using implicit solvent models or reducing the number of iterations in the future. Fourth, our algorithm only used the minimized structures to calculate energy descriptors. Based on molecular dynamics simulations, we could explore the frames in the complete conformational space at reasonable computational costs to reveal novel clues from an energy viewpoint. Fifth, our research illustrated that the subsets of MPDs and MPRs showed different tendencies in terms of energy influences and evaluation metrics. These observations implied that the specific prediction models could be established based on different subsets, which may improve prediction performance. Sixth, the experimentally solved complex structures are rather limited for PNIs but could be expanded using modern modeling techniques. We thus could evaluate our method using the predicted complex structures. Alternatively, pure sequence-based prediction algorithms may be developed. Altogether, the present work provides a comprehensive survey and effective predictors for the effects triggered by MPDs and MPRs, which can facilitate our understanding of the mechanism underlying PNIs.

## Conclusions

In this work, we first made a systematic comparison between the effects of MPDs and MPRs according to the properties of mutated residues, suggesting that these two mutation classes showed specific preferences for binding affinity changes. Accordingly, we constructed two prediction models to separately estimate the binding affinity change values of PDIs and PRIs. Compared with the existing algorithms, we introduced novel energy- and nonenergy-based descriptors and ensemble learning-based architectures to improve prediction accuracy. Although similar frameworks were achieved for MPDs and MPRs, the selected features were different and further revealed the specificity of the two mutation classes. Besides regression models, we developed two classification models that identified mutations remarkably reducing binding affinities. The promising results for diversified datasets illustrated the usefulness and robustness of our algorithm. The PEMPNI webserver could offer a useful computational platform to investigate the effects of mutations on PDIs and PRIs.

## Supporting information

S1 Fig Selection of optimal parameters for energy-based and integrative models. (A) Performance of energy models on MPD276. (B) Performance of integrative models on MPD276 and MPR233.
(PDF)

S2 Fig MPDs and MPRs involved in different types of PNIs and major binding modes. (A) MPDs and MPRs involved in different types of PNIs. (B) MPDs and MPRs involved in major interacting nucleotides. (C) MPDs and MPRs involved in major interacting subunits of nucleotides. (D) MPDs and MPRs involved in major interacting contacts between the target residue and nucleotides.
(PDF)

S3 Fig RMSE values of individual and integrative feature groups for LOCOV. (A) RMSE values of energy feature groups for MPD276. (B) RMSE values of energy feature groups for MPR233. (C) RMSE values of nonenergy feature groups for MPD276 and MPR233. The last row in each figure shows the performance of integrative feature groups.
(PDF)

S4 Fig Performance of combining residue- and interface-based feature groups. (PDF)

S5 Fig Performance of combining ETOR and EPI groups on MPD276. (A) Direct integration model. (B) Weighted sum model.
(PDF)

S6 Fig Correlation between energy- and nonenergy-based predicted affinity changes. (A) PCC value for MPD276. (B) PCC value for MPR233.
(PDF)

S7 Fig PCC values of individual and integrative feature groups for independent testing. (A) PCC values of energy feature groups for MPD48. (B) PCC values of energy feature groups for MPR79. (C) PCC values of nonenergy feature groups for MPD48 and MPR79. The last row in each figure shows the performance of integrative feature groups.
(PDF)

S8 Fig RMSE values of individual and integrative feature groups for independent testing. (A) RMSE values of energy feature groups for MPD48. (B) RMSE values of energy feature groups for MPR79. (C) RMSE values of nonenergy feature groups for MPD48 and MPR79. The last row in each figure shows the performance of integrative feature groups.
(PDF)

S9 Fig PCC values of individual and integrative feature groups for independent testing. (A) PCC values of energy feature groups for P.D.M. (B) PCC values of energy feature groups for P.D.S.I. (C) PCC values of nonenergy feature groups for P.D.M and P.D.S.I. The last row in each figure shows the performance of integrative feature groups.
(PDF)

S10 Fig RMSE values of individual and integrative feature groups for independent testing. (A) RMSE values of energy feature groups for P.D.M. (B) RMSE values of energy feature groups for P.D.S.I. (C) RMSE values of nonenergy feature groups for P.D.M and P.D.S.I. The last row in each figure shows the performance of integrative feature groups.
(PDF)

S11 Fig Performance of combining ETOR and EPI groups on MPD-related test sets (i.e., MPD48, P.D.M, and P.D.S.I). (A-C) Direct integration model. (D-F) Weighted sum model.
(PDF)

S12 Fig Performance of different models on independent testing. (A) Energy feature-based model. (B) Nonenergy feature-based model. (C) Integrative model (PEMPNI).
(PDF)

S13 Fig Results of our classification models for predicting mutations significantly decreasing binding affinities. (A) Results for MPD276. (B) Results for MPD48. (C) Results for P.D.M. (D) Results for P.D.S.I. (E) Results for MPR233. (F) Results for MPR79.
(PDF)

S14 Fig Performance of PEMPNI and other methods on representative protein-RNA complexes. (PDF)

S15 Fig Home page and result page of PEMPNI webserver. (PDF)

S1 Table A summary of our datasets used in this work. (PDF)

S2 Table A summary of energy features used in this work. (PDF)

S3 Table A summary of nonenergy features used in this work. (PDF)

S4 Table A summary of parameters used in our models. (PDF)

S5 Table Comparison between MM/GBSA total energy and EWC. (PDF)

S6 Table Performance of three repetitions with different mutant structures. (PDF)
