# Supplementary material for: Systematic comparison and prediction of the effects of missense mutations on protein-DNA and protein-RNA interactions
Source: PLoS Comput Biol. 2021 Apr 19;17(4):e1008951. doi: 10.1371/journal.pcbi.1008951 (PMC8084330; doi:10.1371/journal.pcbi.1008951)
Supplement: S1 Fig — (A) Performance of energy models on MPD276. (B) Performance of integrative models on MPD276 and MPR233. (PDF) [file pcbi.1008951.s001.pdf]

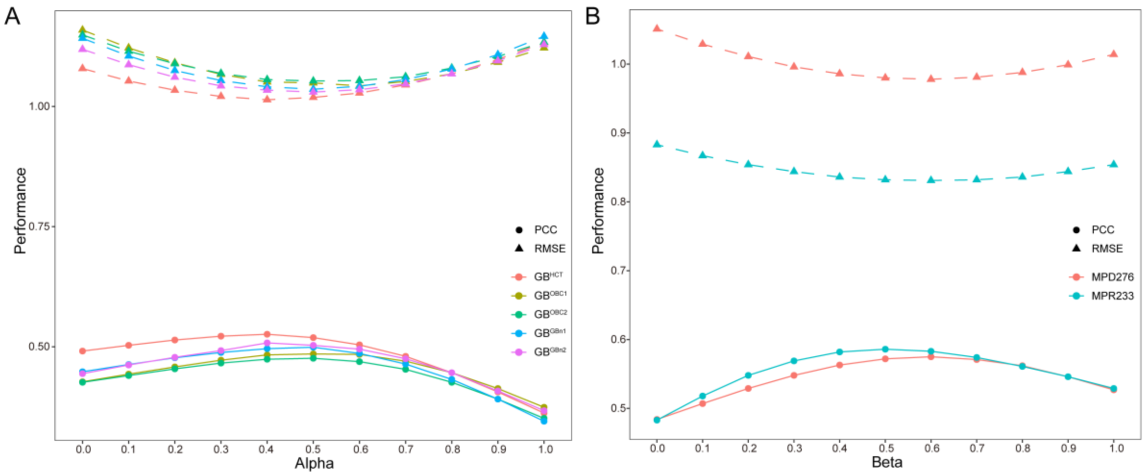

S1 Fig. Selection of optimal parameters for energy-based and integrative models. (A) Performance of energy models on MPD276. (B) Performance of integrative models on MPD276 and MPR233.
