# Supplementary material for: Systematic comparison and prediction of the effects of missense mutations on protein-DNA and protein-RNA interactions
Source: PLoS Comput Biol. 2021 Apr 19;17(4):e1008951. doi: 10.1371/journal.pcbi.1008951 (PMC8084330; doi:10.1371/journal.pcbi.1008951)
Supplement: S2 Fig — (A) MPDs and MPRs involved in different types of PNIs. (B) MPDs and MPRs involved in major interacting nucleotides. (C) MPDs and MPRs involved in major interacting subunits of nucleotides. (D) MPDs and MPRs involved in major interacting contacts between the target residue and nucleotides. (PDF) [file pcbi.1008951.s002.pdf]

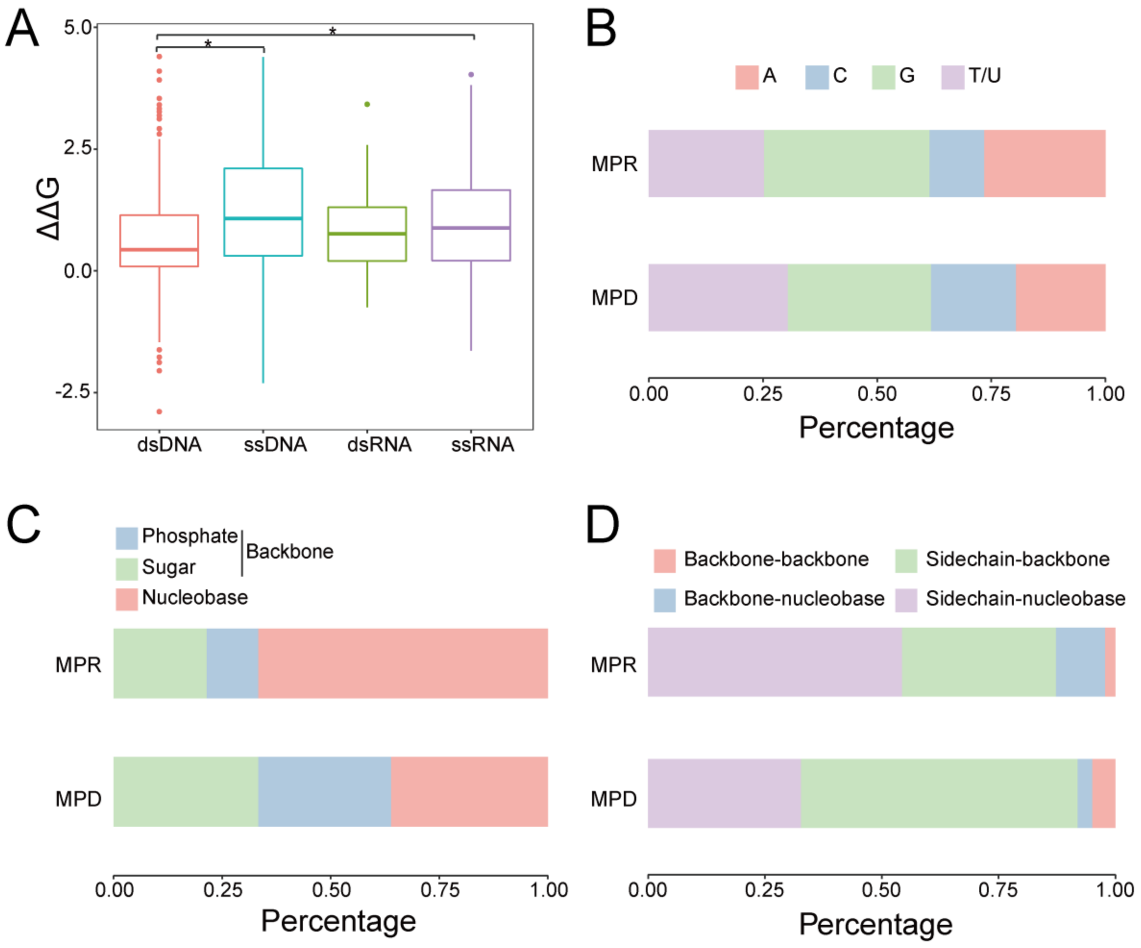


**S2 Fig. MPDs and MPRs involved in different types of PNIs and major binding modes.** (A) MPDs and MPRs involved in different types of PNIs. (B) MPDs and MPRs involved in major interacting nucleotides. (C) MPDs and MPRs involved in major interacting subunits of nucleotides. (D) MPDs and MPRs involved in major interacting contacts between the target residue and nucleotides.
