# Supplementary figures and images for: Systematic comparison and prediction of the effects of missense mutations on protein-DNA and protein-RNA interactions
Source: PLoS Comput Biol. 2021 Apr 19;17(4):e1008951. doi: 10.1371/journal.pcbi.1008951 (PMC8084330; doi:10.1371/journal.pcbi.1008951)

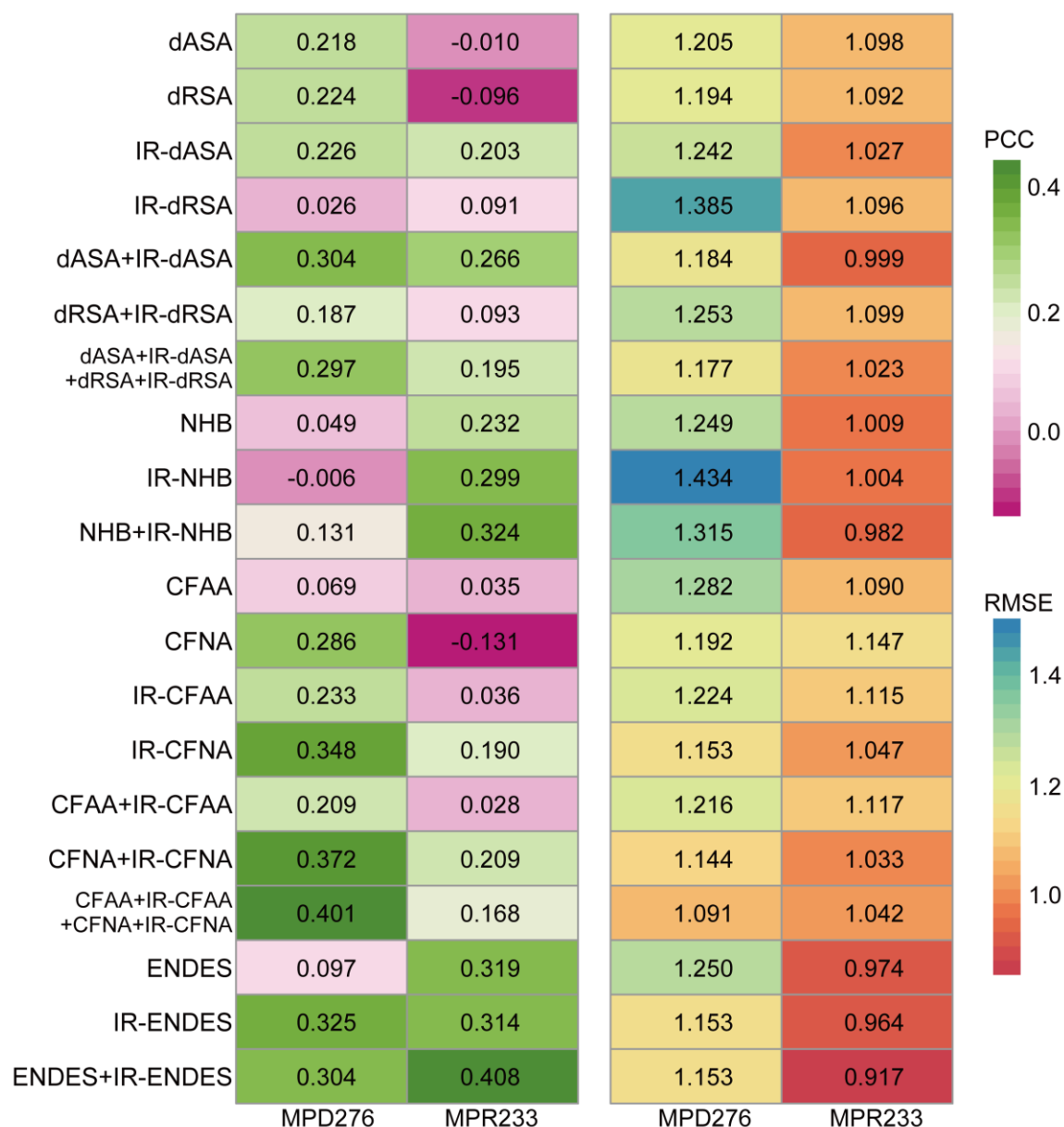

**S4 Fig. Performance of combining residue- and interface-based feature groups.**

Supplement: S4 Fig — (PDF) [file pcbi.1008951.s004.pdf]

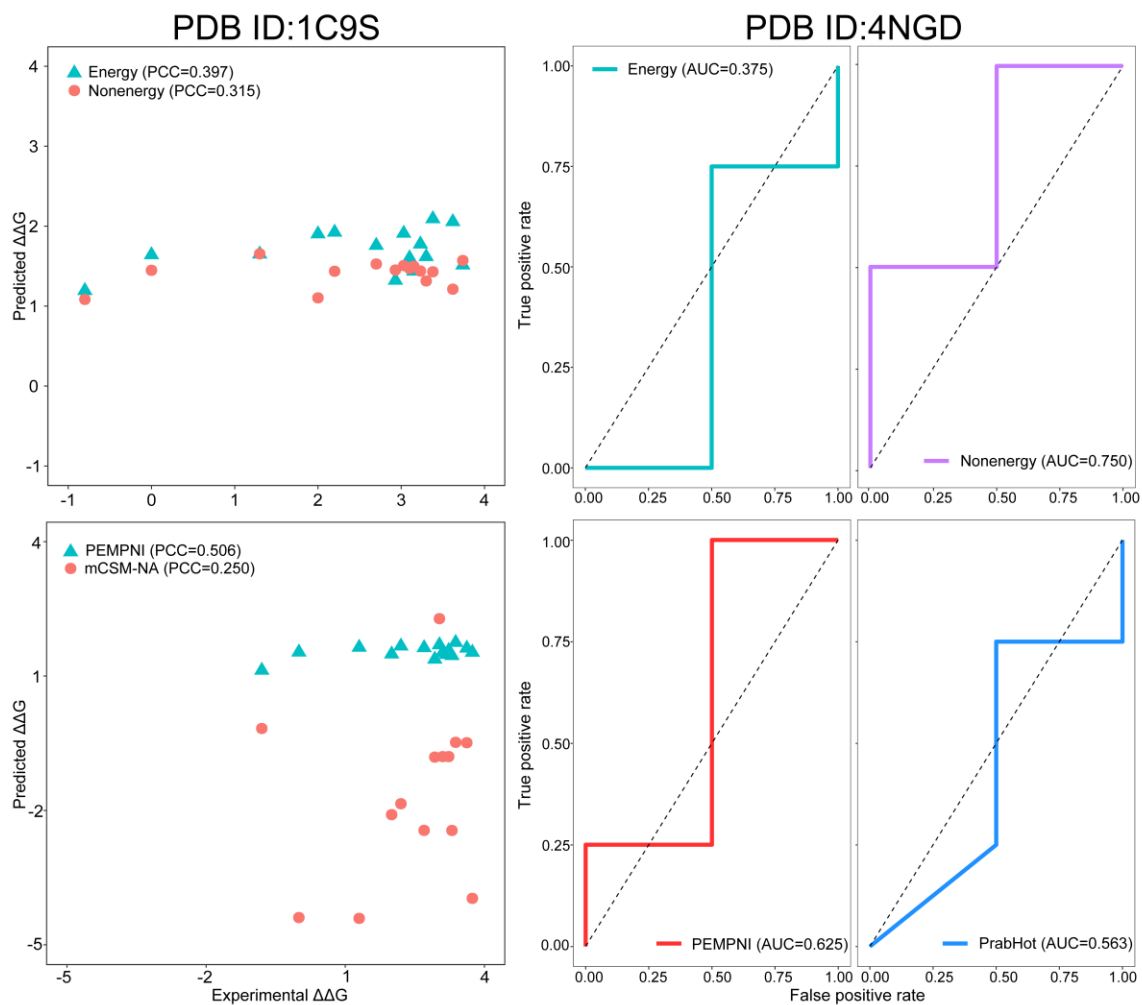

**S14 Fig. Performance of PEMPNI and other methods on representative protein-RNA complexes.**

Supplement: S14 Fig — (PDF) [file pcbi.1008951.s014.pdf]
