# Supplementary material for: Systematic comparison and prediction of the effects of missense mutations on protein-DNA and protein-RNA interactions
Source: PLoS Comput Biol. 2021 Apr 19;17(4):e1008951. doi: 10.1371/journal.pcbi.1008951 (PMC8084330; doi:10.1371/journal.pcbi.1008951)
Supplement: S5 Fig — (A) Direct integration model. (B) Weighted sum model. (PDF) [file pcbi.1008951.s005.pdf]

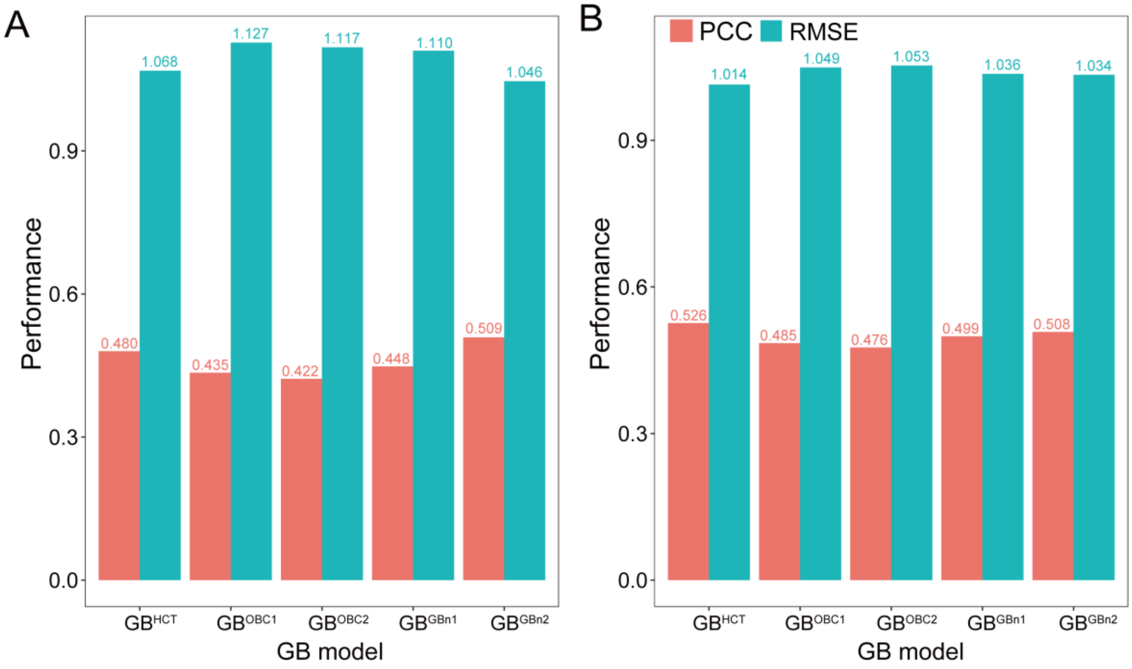

S5 Fig. Performance of combining ETOR and EPI groups on MPD276. (A) Direct integration model. (B) Weighted sum model.
