# Supplementary material for: Systematic comparison and prediction of the effects of missense mutations on protein-DNA and protein-RNA interactions
Source: PLoS Comput Biol. 2021 Apr 19;17(4):e1008951. doi: 10.1371/journal.pcbi.1008951 (PMC8084330; doi:10.1371/journal.pcbi.1008951)
Supplement: S6 Fig — (A) PCC value for MPD276. (B) PCC value for MPR233. (PDF) [file pcbi.1008951.s006.pdf]

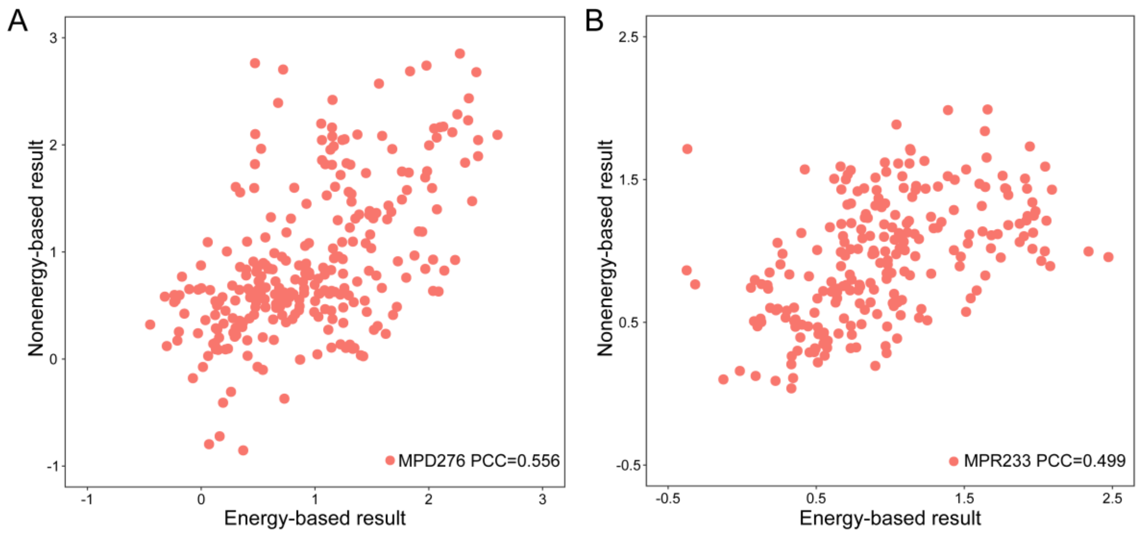

S6 Fig. Correlation between energy- and nonenergy-based predicted affinity changes.

(A) PCC value for MPD276. (B) PCC value for MPR233.
