# Supplementary material for: Systematic comparison and prediction of the effects of missense mutations on protein-DNA and protein-RNA interactions
Source: PLoS Comput Biol. 2021 Apr 19;17(4):e1008951. doi: 10.1371/journal.pcbi.1008951 (PMC8084330; doi:10.1371/journal.pcbi.1008951)
Supplement: S8 Fig — (A) RMSE values of energy feature groups for MPD48. (B) RMSE values of energy feature groups for MPR79. (C) RMSE values of nonenergy feature groups for MPD48 and MPR79. The last row in each figure shows the performance of integrative feature groups. (PDF) [file pcbi.1008951.s008.pdf]

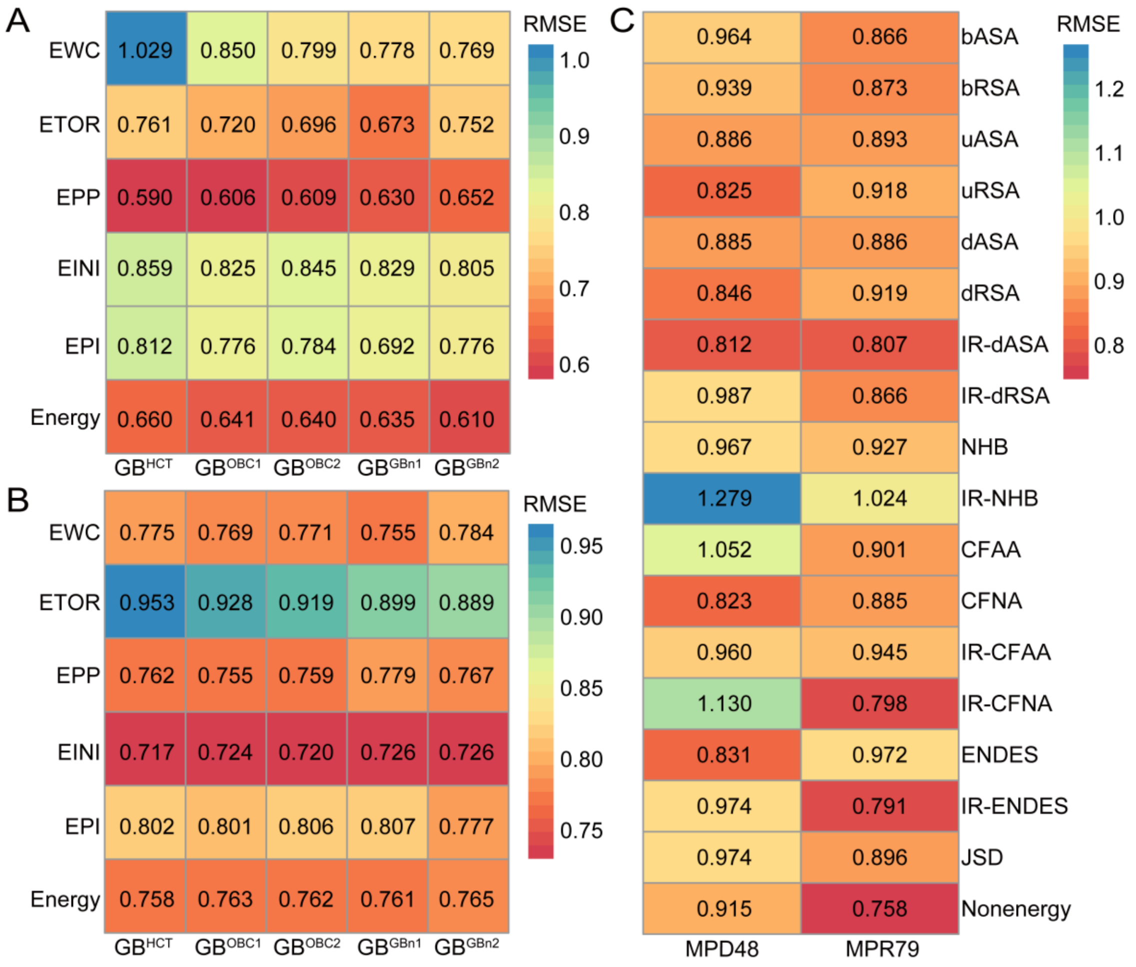

S8 Fig. RMSE values of individual and integrative feature groups for independent testing. (A) RMSE values of energy feature groups for MPD48. (B) RMSE values of energy feature groups for MPR79. (C) RMSE values of nonenergy feature groups for MPD48 and MPR79. The last row in each figure shows the performance of integrative feature groups.
