# Supplementary material for: Systematic comparison and prediction of the effects of missense mutations on protein-DNA and protein-RNA interactions
Source: PLoS Comput Biol. 2021 Apr 19;17(4):e1008951. doi: 10.1371/journal.pcbi.1008951 (PMC8084330; doi:10.1371/journal.pcbi.1008951)
Supplement: S9 Fig — (A) PCC values of energy feature groups for P.D.M. (B) PCC values of energy feature groups for P.D.S.I. (C) PCC values of nonenergy feature groups for P.D.M and P.D.S.I. The last row in each figure shows the performance of integrative feature groups. (PDF) [file pcbi.1008951.s009.pdf]

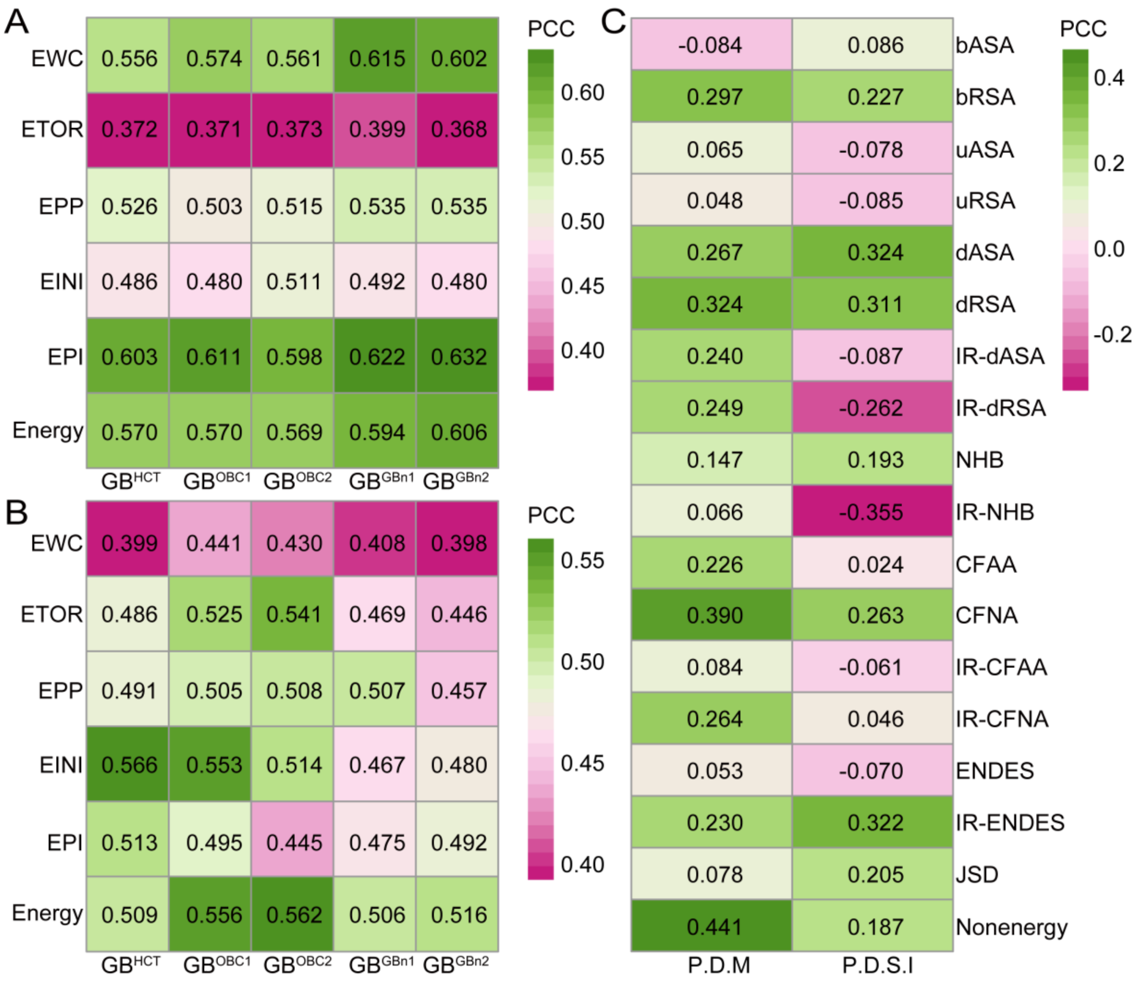

S9 Fig. PCC values of individual and integrative feature groups for independent testing. (A) PCC values of energy feature groups for P.D.M. (B) PCC values of energy feature groups for P.D.S.I. (C) PCC values of nonenergy feature groups for P.D.M and P.D.S.I. The last row in each figure shows the performance of integrative feature groups.
