# Supplementary material for: Systematic comparison and prediction of the effects of missense mutations on protein-DNA and protein-RNA interactions
Source: PLoS Comput Biol. 2021 Apr 19;17(4):e1008951. doi: 10.1371/journal.pcbi.1008951 (PMC8084330; doi:10.1371/journal.pcbi.1008951)
Supplement: S11 Fig — (A-C) Direct integration model. (D-F) Weighted sum model. (PDF) [file pcbi.1008951.s011.pdf]

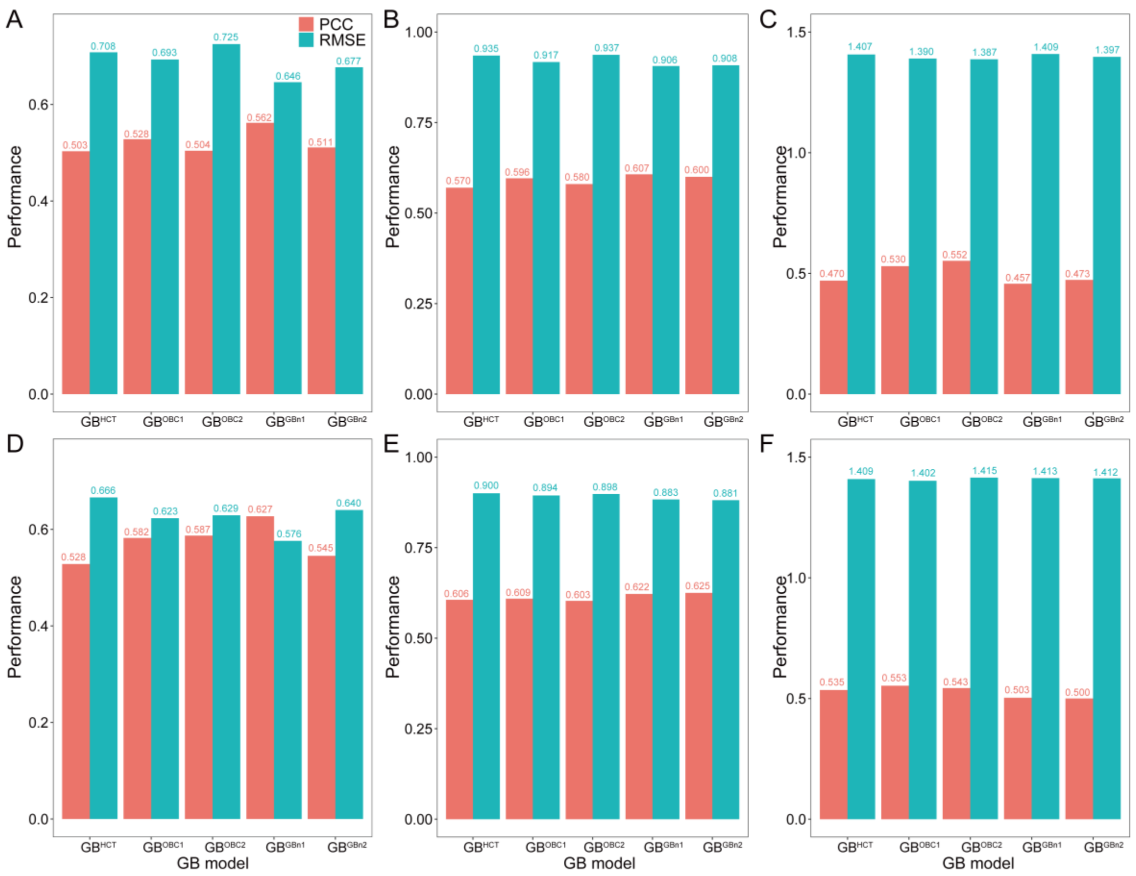

S11 Fig. Performance of combining ETOR and EPI groups on MPD-related test sets (i.e., MPD48, P.D.M, and P.D.S.I). (A-C) Direct integration model. (D-F) Weighted sum model.
