# Supplementary material for: Systematic comparison and prediction of the effects of missense mutations on protein-DNA and protein-RNA interactions
Source: PLoS Comput Biol. 2021 Apr 19;17(4):e1008951. doi: 10.1371/journal.pcbi.1008951 (PMC8084330; doi:10.1371/journal.pcbi.1008951)
Supplement: S12 Fig — (A) Energy feature-based model. (B) Nonenergy feature-based model. (C) Integrative model (PEMPNI). (PDF) [file pcbi.1008951.s012.pdf]

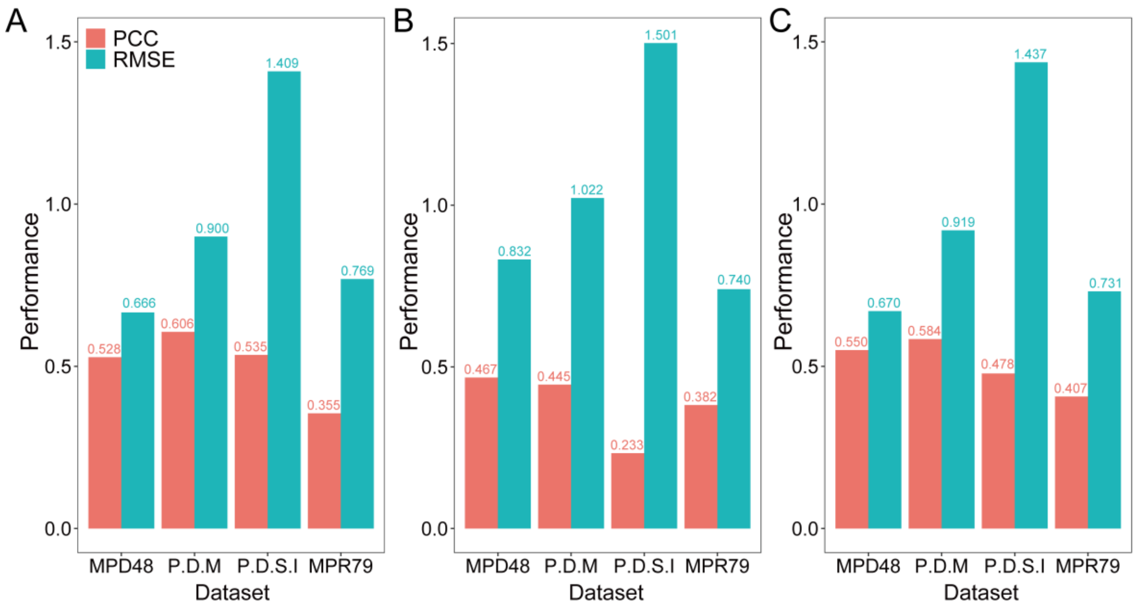

S12 Fig. Performance of different models on independent testing. (A) Energy feature-based model. (B) Nonenergy feature-based model. (C) Integrative model (PEMPNI).
