# Supplementary material for: Systematic comparison and prediction of the effects of missense mutations on protein-DNA and protein-RNA interactions
Source: PLoS Comput Biol. 2021 Apr 19;17(4):e1008951. doi: 10.1371/journal.pcbi.1008951 (PMC8084330; doi:10.1371/journal.pcbi.1008951)
Supplement: S13 Fig — (A) Results for MPD276. (B) Results for MPD48. (C) Results for P.D.M. (D) Results for P.D.S.I. (E) Results for MPR233. (F) Results for MPR79. (PDF) [file pcbi.1008951.s013.pdf]

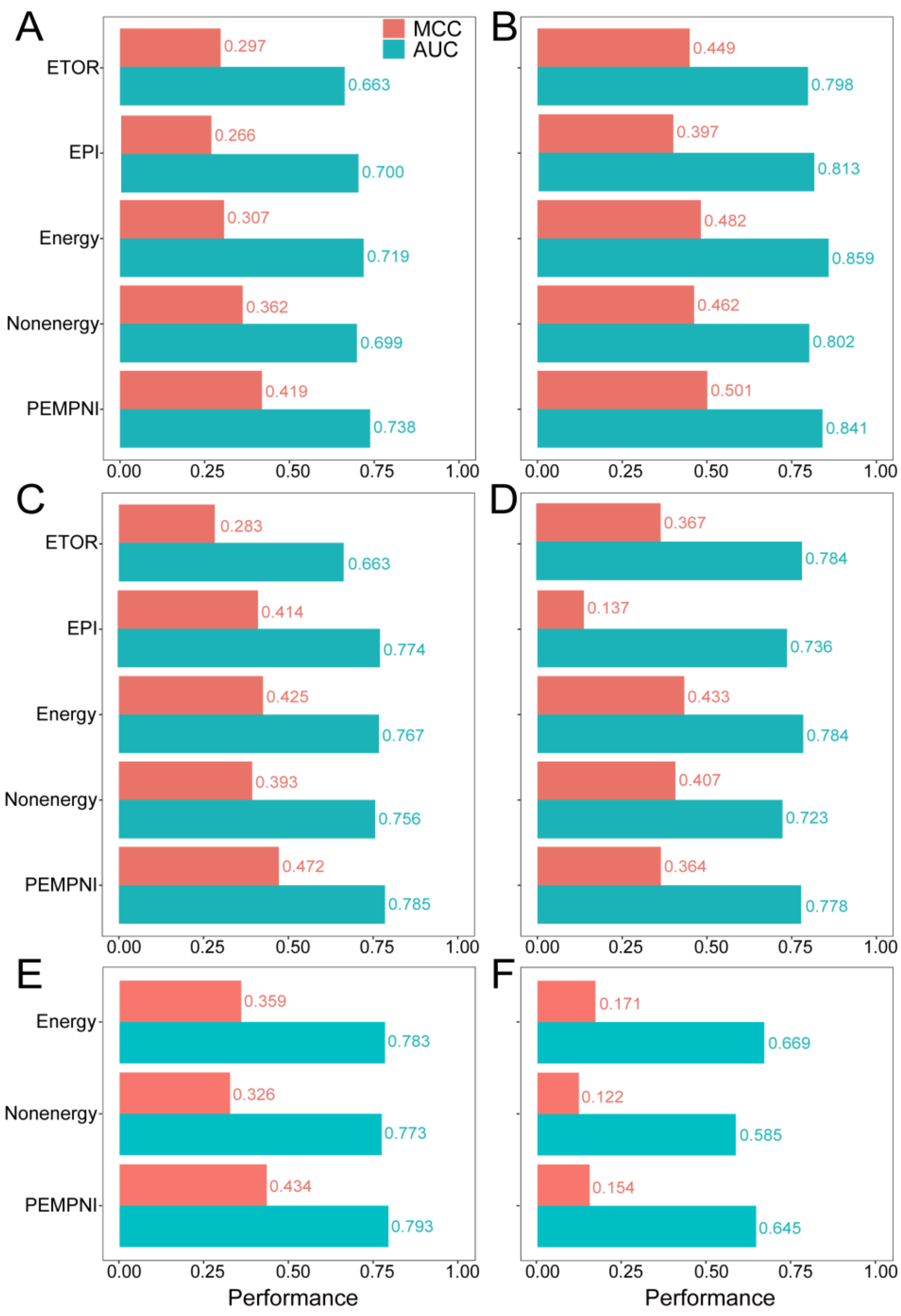

S13 Fig. Results of our classification models for predicting mutations significantly decreasing binding affinities. (A) Results for MPD276. (B) Results for MPD48. (C) Results for P.D.M. (D) Results for P.D.S.I. (E) Results for MPR233. (F) Results for MPR79.
