# Supplementary material for: Systematic comparison and prediction of the effects of missense mutations on protein-DNA and protein-RNA interactions
Source: PLoS Comput Biol. 2021 Apr 19;17(4):e1008951. doi: 10.1371/journal.pcbi.1008951 (PMC8084330; doi:10.1371/journal.pcbi.1008951)
Supplement: S1 Table — (PDF) [file pcbi.1008951.s016.pdf]

S1 Table. A summary of our datasets used in this work

Dataset	Complex	Mutation	ΔΔG<0	ΔΔG=0	ΔΔG>0	ΔΔG<1	ΔΔG≥1
MPD276	53	276	39	0	237	175	101
MPD48	20	48	12	0	36	40	8
Total	73	324	51	0	273	215	109
MPR233	47	233	34	0	199	129	104
MPR79	14	79	9	2	68	53	26
Total	61	312	43	2	267	182	130

$\Delta\Delta G=\Delta G^{MT}-\Delta G^{WT}$
