# Supplementary material for: Systematic comparison and prediction of the effects of missense mutations on protein-DNA and protein-RNA interactions
Source: PLoS Comput Biol. 2021 Apr 19;17(4):e1008951. doi: 10.1371/journal.pcbi.1008951 (PMC8084330; doi:10.1371/journal.pcbi.1008951)
Supplement: S2 Table — (PDF) [file pcbi.1008951.s017.pdf]

S2 Table. A summary of energy features used in this work

Feature group	Dimensionality	Description
EWC	10 (5+5)	Energies of the whole complex denoted by five descriptors (i.e., ΔG, ΔE_{ele}, ΔE_{vdw}, ΔG_{GB}, and ΔG_{SA}).
ETOR	60 (5*6+5*6)	Energies between the target and other residues (from six partitions).
EPP	60 (5*6+5*6)	Energies of the partitioned protein (six partitions).
EINI	20 (5*2+5*2)	Energies of the interface and noninterface (two partitions).
EPI	30 (5*3+5*3)	Energies of the partitioned interface (three partitions).

The features extracted from the wild-type complex and the differences in measures of the original and mutant complexes were used in this work.
