# Supplementary material for: Systematic comparison and prediction of the effects of missense mutations on protein-DNA and protein-RNA interactions
Source: PLoS Comput Biol. 2021 Apr 19;17(4):e1008951. doi: 10.1371/journal.pcbi.1008951 (PMC8084330; doi:10.1371/journal.pcbi.1008951)
Supplement: S3 Table — (PDF) [file pcbi.1008951.s018.pdf]

S3 Table. A summary of nonenergy features used in this work

Property	Feature group	Dimensionality	Description
Solvent accessibility	bASA	10 (5+5)	Absolute solvent accessibilities of the target residue in the bound state based on five atomic groups (i.e., total atoms, mainchain atoms, sidechain atoms, polar sidechain atoms, and nonpolar sidechain atoms).
	bRSA	10 (5+5)	Relative solvent accessibilities of the target residue in the bound state.
	uASA	10 (5+5)	Absolute solvent accessibilities of the target residue in the unbound state.
	uRSA	10 (5+5)	Relative solvent accessibilities of the target residue in the unbound state.
	dASA	10 (5+5)	Differences in absolute solvent accessibilities of the target residue between the two states.
	dRSA	10 (5+5)	Differences in relative solvent accessibilities of the target residue between the two states.
	IR-dASA	10 (5+5)	Cumulative dASA values for interface residues.
	IR-dRSA	10 (5+5)	Cumulative dRSA values for interface residues.
Hydrogen bond	NHB	2 (1+1)	Number of hydrogen bonds between the target residue and the rest of a complex.
	IR-NHB	2 (1+1)	Cumulative NHB values for interface residues.
Contact feature	CFAA	4 (2+2)	Residue-residue contact strength and average atomic contact strength between the target residue and amino acids.

	CFNA	4 (2+2)	Residue-residue contact strength and average atomic contact strength between the target residue and nucleic acids.
	IR-CFAA	4 (2+2)	Cumulative CFAA values for interface residues.
	IR-CFNA	4 (2+2)	Cumulative CFNA values for interface residues.
ENDES feature	ENDES	14 (7+7)	Seven knowledge-based scores of the target residue.
	IR-ENDES	14 (7+7)	Cumulative ENDES values for interface residues.
Evolutionary conservation	JSD	2 (1+1)	Jensen-Shannon divergence of the target residue.

The features extracted from the wild-type complex and the differences in measures of the original and mutant complexes were used in this work.
