# Supplementary material for: Systematic comparison and prediction of the effects of missense mutations on protein-DNA and protein-RNA interactions
Source: PLoS Comput Biol. 2021 Apr 19;17(4):e1008951. doi: 10.1371/journal.pcbi.1008951 (PMC8084330; doi:10.1371/journal.pcbi.1008951)
Supplement: S4 Table — (PDF) [file pcbi.1008951.s019.pdf]

S4 Table. A summary of parameters used in our models

GB model	Alias	Radii	Dielectric constant	Calculation of the polar solvation energy	Method for calculation of the nonpolar solvation energy			
					SASA	ΔG_{SA}	γ	b
GB^{HCT}	GB1	mbondi	1.0	GB^{HCT}	LCPO	γSASA+b	0.0072	0.0
GB^{OBC1}	GB2	mbondi2	1.0	GB^{OBC} model I	LCPO	γSASA+b	0.0050	0.0
GB^{OBC2}	GB5	mbondi2	1.0	GB^{OBC} model II	LCPO	γSASA+b	0.0050	0.0
GB^{GBn1}	GB7	bondi	1.0	GB^{GBn} model I	LCPO	γSASA+b	0.0050	0.0
GB^{GBn2}	GB8	mbondi3	1.0	GB^{GBn} model II	LCPO	γSASA+b	0.0050	0.0

Weight	Class	GB^{HCT}	GB^{OBC1}	GB^{OBC2}	GB^{GBn1}	GB^{GBn2}
α	MPD	0.4	0.5	0.5	0.5	0.4
β	MPD	0.6	-	-	-	-
β	MPR	-	0.5	-	-	-
