# Supplementary material for: Systematic comparison and prediction of the effects of missense mutations on protein-DNA and protein-RNA interactions
Source: PLoS Comput Biol. 2021 Apr 19;17(4):e1008951. doi: 10.1371/journal.pcbi.1008951 (PMC8084330; doi:10.1371/journal.pcbi.1008951)
Supplement: S5 Table — (PDF) [file pcbi.1008951.s020.pdf]

## S5 Table. Comparison between MM/GBSA total energy and EWC

| | $GB^{HCT}$ | | $GB^{OBC1}$ | | $GB^{OBC2}$ | | $GB^{GBn1}$ | | $GB^{GBn2}$ | |
|---|---|---|---|---|---|---|---|---|---|---|
| | PCC | RMSE | PCC | RMSE | PCC | RMSE | PCC | RMSE | PCC | RMSE |
| MM/GBSA (MPD276) | 0.306 | 1.400 | 0.300 | 1.300 | 0.373 | 1.337 | 0.285 | 1.363 | 0.141 | 1.478 |
| EWC (MPD276) | 0.274 | 1.325 | 0.261 | 1.322 | 0.282 | 1.325 | 0.283 | 1.292 | 0.196 | 1.353 |
| MM/GBSA (MPR233) | 0.182 | 1.245 | -0.022 | 1.259 | 0.063 | 1.183 | -0.035 | 1.194 | -0.037 | 1.163 |
| EWC (MPR233) | 0.508 | 0.867 | 0.527 | 0.855 | 0.524 | 0.857 | 0.489 | 0.878 | 0.481 | 0.885 |
