# Supplementary material for: Systematic comparison and prediction of the effects of missense mutations on protein-DNA and protein-RNA interactions
Source: PLoS Comput Biol. 2021 Apr 19;17(4):e1008951. doi: 10.1371/journal.pcbi.1008951 (PMC8084330; doi:10.1371/journal.pcbi.1008951)
Supplement: S6 Table — (PDF) [file pcbi.1008951.s021.pdf]

S6 Table. Performance of three repetitions with different mutant structures

	GB^{HCT} (MPD276)				GB^{OBC1} (MPR233)			
	PCC		RMSE		PCC		RMSE	
EWC	0.282±0.012	0.274	1.314±0.014	1.325	0.502±0.021	0.527	0.869±0.012	0.855
ETOR	0.354±0.009	0.363	1.135±0.005	1.132	0.072±0.018	0.054	1.064±0.010	1.076
EPP	0.456±0.009	0.467	1.063±0.006	1.056	0.423±0.003	0.422	0.917±0.003	0.919
EINI	0.420±0.008	0.424	1.123±0.006	1.116	0.286±0.002	0.287	0.975±0.001	0.976
EPI	0.497±0.011	0.485	1.070±0.012	1.083	0.260±0.004	0.261	0.996±0.004	0.999
Energy	0.442±0.008	0.446	1.092±0.011	1.086	0.457±0.010	0.469	0.902±0.004	0.898

The average measures and standard deviations are reported for the three repetitions. Meanwhile, the major results in the manuscript are also shown as the reference. Energy denotes the combination of all energy features.
